# Stem cell and neurogenic gene-expression profiles link prostate basal cells to aggressive prostate cancer

**DOI:** 10.1038/ncomms10798

**Published:** 2016-02-29

**Authors:** Dingxiao Zhang, Daechan Park, Yi Zhong, Yue Lu, Kiera Rycaj, Shuai Gong, Xin Chen, Xin Liu, Hsueh-Ping Chao, Pamela Whitney, Tammy Calhoun-Davis, Yoko Takata, Jianjun Shen, Vishwanath R. Iyer, Dean G. Tang

**Affiliations:** 1 Department of Epigenetics and Molecular Carcinogenesis, University of Texas MD Anderson Cancer Center, Smithville, 78957 Texas, USA; 2 Department of Molecular Biosciences, Institute for Cellular and Molecular Biology, Center for Systems and Synthetic Biology, University of Texas at Austin, Austin, 78712 Texas, USA; 3 Cancer Stem Cell Institute, Research Center for Translational Medicine, East Hospital, Tongji University School of Medicine, 2001120 Shanghai, China; 4 Centers for Cancer Epigenetics, Stem Cell and Developmental Biology, RNA Interference and Non-Coding RNAs and Molecular Carcinogenesis, University of Texas MD Anderson Cancer Center, Houston, 77030 Texas, USA

## Abstract

The prostate gland mainly contains basal and luminal cells constructed as a pseudostratified epithelium. Annotation of prostate epithelial transcriptomes provides a foundation for discoveries that can impact disease understanding and treatment. Here we describe a genome-wide transcriptome analysis of human benign prostatic basal and luminal epithelial populations using deep RNA sequencing. Through molecular and biological characterizations, we show that the differential gene-expression profiles account for their distinct functional properties. Strikingly, basal cells preferentially express gene categories associated with stem cells, neurogenesis and ribosomal RNA (rRNA) biogenesis. Consistent with this profile, basal cells functionally exhibit intrinsic stem-like and neurogenic properties with enhanced rRNA transcription activity. Of clinical relevance, the basal cell gene-expression profile is enriched in advanced, anaplastic, castration-resistant and metastatic prostate cancers. Therefore, we link the cell-type-specific gene signatures to aggressive subtypes of prostate cancer and identify gene signatures associated with adverse clinical features.

## Abstract (web summary)

Gene-expression profiles can be used to predict the prognosis of cancer patients. Here, the authors describe gene expression profiles of human prostate epithelial lineages and show that basal cells have intrinsic stem and neurogenic properties, and molecularly resemble aggressive prostate cancer.

## Graphical abstract

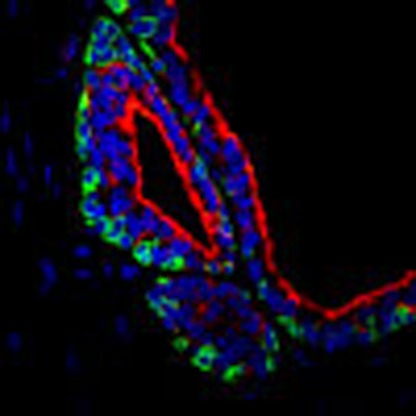

Prostate cancer (PCa) is a heterogeneous malignancy harbouring phenotypically and functionally diverse subpopulations of cancer cells^1^,^2^. To better understand PCa cell heterogeneity, it is crucial to dissect the biology of normal prostate epithelial lineages, which could help address important questions such as the cell(s)-of-origin of PCa. The prostate is an exocrine gland in which prostatic ducts are lined by three cell types: secretory luminal cells, basal cells and rare neuroendocrine cells^3^. Developmentally, the murine prostate originates from an ancestral p63^+^AR^−^ basal stem cell (SC) population^4^. Prostate regeneration assays also reveal SCs with multi-lineage differentiation potential to be localized to the basal layer of the mouse prostate^5^,^6^,^7^,^8^. Lineage-tracing studies, on the other hand, suggest that both basal and luminal cell layers in adult murine prostate contain lineage-restricted stem/progenitor cells^9^,^10^ although primitive SCs reside in the basal layer^10^. In support, some mouse prostate basal cells can undergo asymmetric divisions (a cardinal feature of SCs), whereas luminal cells only undergo symmetrical divisions^11^. In the human prostate, there is also evidence that the basal cell layer harbours regenerative SCs^6^,^12^. Nevertheless, ‘direct' evidence is still lacking, as, for obvious reasons, lineage tracing cannot be performed in the live human prostate.

Defining the cells-of-origin for cancer is of great value for patient tumour stratification and delivering personalized treatment. Luminal cells are traditionally believed to be the cell-of-origin for human PCa due to the predominantly luminal-like phenotype of the disease. However, tissue regeneration-based assays indicate that only a subset of basal cells can function as the cell-of-origin for PCa^6^, whereas studies in genetic mouse models show that PCa can originate from both basal and luminal cell lineages and that luminal cells are even more susceptible to tumourigenesis^9^,^13^. It is presently unclear what might account for the discrepancies in these two lines of studies. Potentially, an in-depth understanding of the gene-expression differences in normal human prostate basal versus luminal cells could help illuminate the intrinsic functional differences between the two cell types, which, in turn, could offer fresh insights into the cell-of-origin for (different types of) PCa.

Gene expression is a key determinant of cellular phenotypes. A comprehensive annotation of the transcriptome would facilitate a better understanding of how gene expression influences phenotypic manifestations. Recently, RNA sequencing (RNA-Seq) has been widely used to delineate the entire transcriptome in a large variety of tissues and cancers at unprecedented depth and sensitivity. In particular, deep RNA-Seq allows the detection of the novel and relatively low abundant transcripts (for example, long non-coding RNAs). Comprehensive exploration of the DNA mutational landscape of PCa has been achieved using genome-wide sequencing^14^,^15^. Recent TCGA project also includes the RNA-Seq data for hundreds of PCa patients. However, all large-scale sequencing studies as of yet in the field have used heterogeneous tissue pieces (which contain epithelial and non-epithelial cells) as the material for DNA and RNA extraction, suggesting a lack of insight into the biology of distinct epithelial lineages.

Here we describe a detailed transcriptome analysis of unperturbed human benign prostatic basal and luminal cells by deep RNA-Seq. The results reveal the surprising findings that basal cells are intrinsically enriched in gene sets normally associated with SCs, neurogenesis and ribosomal RNA (rRNA) biogenesis. We show that, coupled with their unique gene-expression profiles, basal cells functionally exhibit intrinsic stem-like and neurogenic properties with enhanced rRNA transcription activity. We further link the basal cell gene signature to those in aggressive, castration-resistant and anaplastic PCa subtypes. We also identify molecular signatures associated with patient outcome. Altogether, our results provide the most functionally comprehensive study on, and a resource of the transcriptomes in, unperturbed subtypes of human prostatic epithelial cells that shed light on PCa aetiology.

## Results

### Distinct transcriptomes of prostatic basal and luminal cells

**Figure 1:**
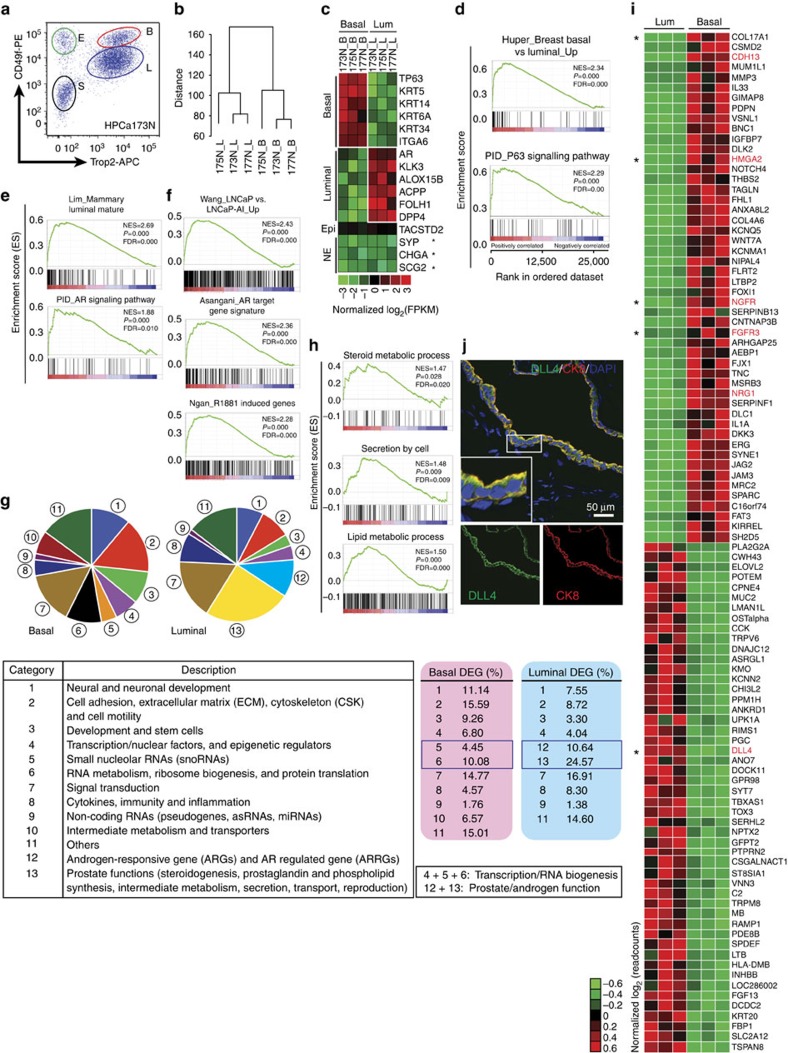
Distinct gene-expression profiles of prostatic basal and luminal cells. (**a**) FACS plots of prostate basal (B), luminal (L), endothelial-enriched (E) and stromal-enriched (S) populations identified as Trop2^+^CD49f^hi^, Trop2^+^CD49f^lo^, Trop2^-^cd49f^hi^ and Trop2^-^CD49f^-^, respectively. (**b**) Hierarchical clustering of RNA-Seq data in three pairs of benign prostatic basal (N_B) and luminal (N_L) cells. All detected genes were used in the clustering analysis. *y*-axis shows euclidean distance for log2 (normalized read counts). (**c**) Heatmap presentation of expression of known phenotypic markers corresponding to different prostate cell lineages. Asterisks indicates the genes expressed at very low levels (FPKM<0.18). (**d**–**f**) Representative GSEA results in basal (**d**) and luminal (**e**) cells. In (**f**), androgen-responsive genes and AR-regulated genes in the three indicated data sets are enriched in luminal cells. (**g**) Distinct transcriptomic profiles of human prostatic basal and luminal cells. Shown are pie charts of gene categories (Supplementary Data 1) over-represented in basal and luminal populations. Descriptions of each functional category are given in the table below. Percentages of each category in basal versus luminal cells are marked in red and blue, respectively (below, right). (**h**) GSEA results for the enrichment of indicated gene signatures in luminal cells. (**i**) Heatmap of the top 50 putative marker genes in each epithelial lineage. Asterisks indicate the genes whose exclusive cellular localization has been confirmed by immunofluorescence. Genes with their biological functions investigated in this study are coloured in red. See the ‘Methods' section for detail. (**j**) Immunofluorescence of DLL4 and CK8 in benign prostate tissues. Scale bars, 50 μm.

To comprehensively interrogate the molecular features of human prostate epithelial lineages, we determined the transcriptomes of unperturbed benign basal and luminal populations by deep paired-end RNA-Seq. Three representative human benign samples (HPCa173N, 175N and 177N) from PCa patients who had largely benign biopsies (Supplementary Fig. 1a and Supplementary Table 1) were selected for purification of basal (Trop2^+^CD49f^hi^) and luminal (Trop2^+^CD49f^lo^) fractions^6^ using fluorescence-activated cell sorting (FACS; Fig. 1a and Supplementary Fig. 1b). Three pairs of total RNAs derived from basal and luminal populations were generated for library preparations and subsequent RNA-Seq.

By deep sequencing of the rRNA-depleted total RNAs, we obtained an average of 211.5 million reads per sample (range from 196.6 to 229.9) with an average mapping rate of 91.7% to the reference human genome (UCSC version hg19; Supplementary Fig. 1c). Unsupervised hierarchical clustering showed that the basal and luminal populations were grouped together and well-separated (Fig. 1b) and MA plot indicated appropriate normalization of our RNA-Seq data (Supplementary Fig. 1d). By applying a stringent statistic threshold of greater than equal to twofold change (FC) and false discovery rate (FDR) of <0.05, we identified a consensus of 853 differentially expressed genes (DEGs) upregulated in basal and 940 DEGs in luminal cells (Supplementary Data 1). Basal and luminal cells showed exclusively high expression in molecules known to be restricted to each cell type (Fig. 1c). As expected, no difference was found for *TACSTD2* (*Trop2*), and neuroendocrine markers showed little expression (fragments per kilobase of exon per million fragments mapped (FPKM)<0.18) in and no difference between the two populations (Fig. 1c).

Gene set enrichment analysis (GSEA) revealed exclusive enrichment patterns of gene signatures related to basal (mammary basal cell signature and p63 pathway, Fig.1d) and luminal (mammary luminal cell signature and AR pathway, Fig. 1e) epithelial phenotypes, respectively. Remarkably, when we extracted three sets of androgen-responsive genes and AR-regulated genes^16^,^17^,^18^, we found that they were all enriched in our luminal cells (Fig. 1f). To further dissect the transcriptomic profiles, we performed the pathway/network enrichment analysis by GSEA and Ingenuity Pathway Analysis (IPA). Strikingly, GSEA revealed that genes upregulated in basal or luminal cells were enriched in markedly different functional pathways, which were corroborated by IPA (Supplementary Data 2 and Supplementary Fig. 1e). Finally, we combined gene ontology (GO) analysis and literature-based manual curation to classify each DEG in each cell type into non-redundant functional categories (Supplementary Data 1). Such thorough gene annotations demonstrated that, consistent with global GSEA and IPA, SCs and development, neural/neuronal development, the cell adhesion and motility, RNA metabolism and ribosome/translation represented the top gene categories in basal cells (Fig. 1g). In contrast, genes upregulated in luminal cells fell into categories of prostate organ function (for example, steroid hormone processing, secretion, lipid metabolism), androgen and AR signalling, and inflammation and immunity (Fig. 1g and Supplementary Fig. 1e). In support, gene signatures corresponding to above categories were highly enriched in luminal cells (Fig. 1e–h). Therefore, our RNA-Seq-derived gene-expression profiles in basal and luminal cells mirror their well-established biological functions and support basal cells as undifferentiated stem-like and luminal cells as differentiated functional cell types. The current study presents the deepest annotated transcriptomes of the two human prostate epithelial lineages.

Interestingly, in addition to coding RNAs, we also identified many differentially expressed non-coding RNAs. Consistent with the reliability of our RNA-Seq data, we observed many AR-regulated ncRNAs (including miRNAs and lncRNAs) in luminal cells and p63-regulated ncRNAs in basal cells (Supplementary Data 1). The functional significance of these changes in regulating human prostate epithelial biology is currently under investigation.

### Identification of novel prostate epithelial lineage markers

In addition to capturing the global view of prostatic basal and luminal cell transcriptomes, our RNA-Seq analysis identified sets of genes specific to each cell type, potentially providing a battery of novel markers that can be used to distinguish the two epithelial lineages. For example, consistent with RNA-Seq data, immunohistochemistry analysis of EGFR and SNAI2 showed enhanced staining intensity and frequency in basal versus luminal cells, whereas CTNNB1 exhibited no staining difference in both layers (Supplementary Fig. 1f,g). We identified the top 50 putative marker genes for each lineage (Fig. 1i) inferred from transcriptomes based on both relative differential expression (FC) and absolute expression levels (normalized read counts; see the ‘Methods' section), some of which were confirmed by immunofluorescence, including COL17A1 and HMGA2 (Supplementary Fig. 1h), DLL4 (Fig. 1j), and FGFR3 and NGFR (see below). In particular, DLL4 was confirmed to be a *bona fide* luminal marker. Considering that NOTCH receptors (NOTCH1/4) were preferentially expressed in basal cells, this may represent an example of signalling crosstalk between basal and luminal cells. Notably, we also identified many transcription and nuclear factors whose expression profiles were strongly linked to each cell type (Supplementary Data 1).

### Intrinsic SC and EMT properties of prostatic basal cells

**Figure 2:**
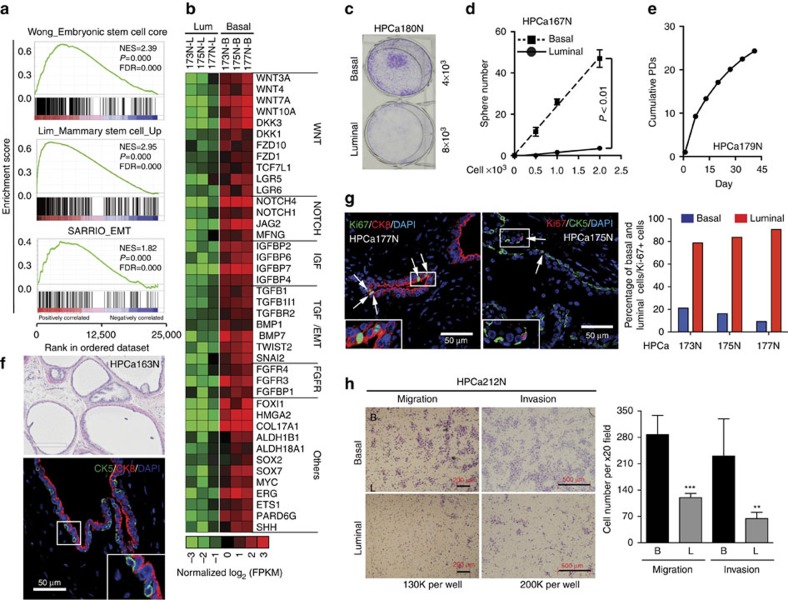
SC and EMT properties of human prostatic basal cells. (**a**) GSEA showing enrichment of SC and EMT gene signatures in basal cells. (**b**) Heatmap of representative SC-associated genes overexpressed in basal cells. (**c**–**e**) Basal cells exhibit high stem/progenitor activities *in vitro*. Shown are colony formation, (**c**) limiting dilution sphere assay, (**d**) and cumulative population doubling (PDs) (**e**) of basal cells derived from one, but a different, patient sample respectively. Results shown (HPCa179N) were representative data of 3 repeat experiments in different patient-derived cell populations. (**f**) Hematoxylin and eosin staining and immunofluorescence of CK5 and CK8 in prostate tissue regenerated *in vivo* from HPCa163N primary basal cells co-injected with mouse UGSM. (**g**) Immunofluorescence of Ki-67, CK8 and CK5 in human benign prostate tissues (left) and quantification of % Ki-67^+^ cells according to lineage identity (right). (**h**) Migration and invasion assays in basal and luminal cells freshly purified from HPCa212N. Representative low-magnification images (left) and quantification data (right) are shown. Data represent means±s.d. from cell number counting of 5–6 random high magnification (× 20) images. Results shown (HPCa212N) were representative data of at least 2–3 repeat experiments in different patient-derived cell populations. The *P* value was calculated using Student's *t*-test **P<0.01 and ***P<0.001. Boxed regions are enlarged. The white arrows indicate the cells stained positive for Ki-67. Scale bars, 50 μm; 200 μm; 400 μm; 500 μm (**f** bottom and **g**; **h** left, migration; **f** top; and **h** right, invasion, respectively).

The unique gene- (for example, SC- and epithelial–mesenchymal transition (EMT)-enriched) expression profile in basal cells (Supplementary Fig. 1e) and current inability to culture fully differentiated AR^+^/PSA^+^ luminal cells prompted us to focus our subsequent studies on basal cells. Freshly purified primary basal cells and short-term expanded cultures (<P3) were utilized in a spectrum of *in vitro* and *in vivo* assays to characterize the basal cell stem/progenitor activities. Our RNA-Seq suggests a stem-like transcriptional programme in basal cells, and many of the known SC-related genes and pathways were upregulated in basal cells (Fig. 2a,b). Consistent with the SC-enriched gene profiles, freshly purified basal cells exhibited much higher clonal (colony formation; Fig. 2c) and clonogenic (sphere-forming; Fig. 2d) capacities than matched luminal cells. Primary basal cells also possessed long-term proliferative capacity (Fig. 2e), as supported by high Ki-67^+^ labelling efficiency (Supplementary Fig. 2a). Importantly, freshly purified basal cells could differentiate into CK8^+^ luminal cells in sphere cultures in the presence of dihydrotestosterone (Supplementary Fig. 2b). The spheres initially emerged as a solid ball and then developed into hollow organoids with secretion inside the lumen. Structurally, p63^+^ and CK5^+^ basal cells mainly resided in the peripheral layer (Supplementary Fig. 2b), consistent with a recent report^19^. Significantly, primary basal cells, when implanted with embryonic urogenital sinus mesenchyme subcutaneously, were able to regenerate glandular structures with luminal differentiation (Fig. 2f and Supplementary Fig. 2c). The basal identity of the cells injected and human origin of the recombinants were verified by p63 and human-specific mitochondria staining, respectively (Supplementary Fig. 2d). Since adult tissue SCs normally remain quiescent *in situ*, we performed double immunofluorescence of Ki-67 with CK5 or CK8 and quantified Ki-67^+^ cells according to epithelial identity. Consistent with observations in the murine prostate^20^, more than 80% of Ki-67^+^ cells were luminal, documenting the relative quiescence of basal cells (Fig. 2g).

Rapid adhesion to collagen and preferential expression of cell-surface integrins have been exploited to enrich SCs in the human prostate^12^. Consistently, basal cells preferentially expressed many genes associated with cell adhesion/cytoskeleton/extracellular matrix remodelling (Fig. 1g). In contrast, luminal cells expressed only about half as many genes that fall into this category (8.7 versus 15.6%). GSEA and IPA indicated that many of cell junction and movement pathways were preferentially enriched in basal cells (Supplementary Fig. 1e and Supplementary Data 2), suggesting that basal cells might be more motile. In support, basal cells expressed an EMT signature and many typical EMT inducers and markers (Fig. 2a,b), and primary basal cells exhibited high migratory and invasive capacities compared with luminal cells (Fig. 2h and Supplementary Fig. 2e).

### Pathways regulating the SC properties of basal cells

**Figure 3:**
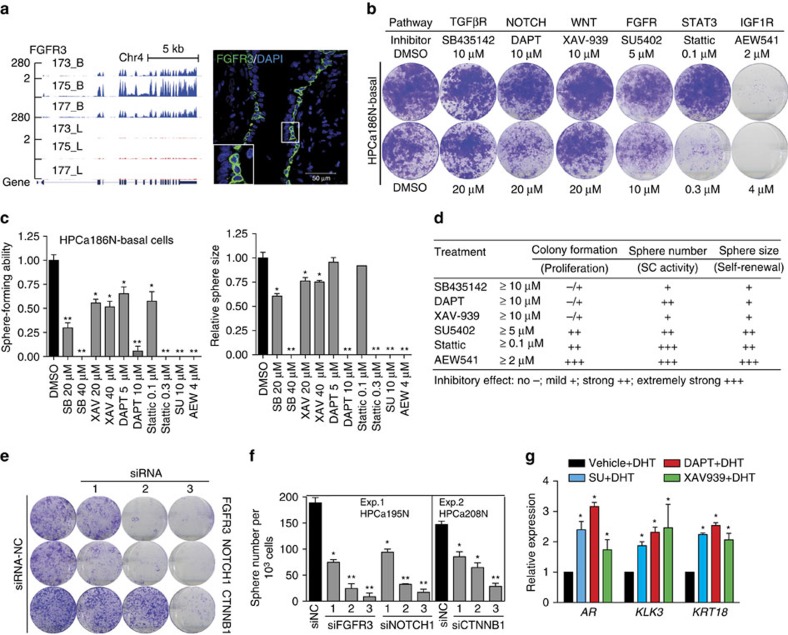
Signalling pathways that regulate human prostatic basal stem/progenitor cell activity. (**a**) Genome-browser view of RNA-Seq signals in *FGFR3* gene region (left) and immunofluorescence of FGFR3 in HPCa177N (right). (**b**,**c**) Effects of select pathway inhibitors on colony formation (**b**), and sphere-forming efficiency and sphere size of basal cells (**c**). Data in **c** was presented as the relative values of treatment groups normalized to vehicle control groups. (**d**) Summary of the data from **b** and **c**, and Supplementary Fig. 3d,e. (**e**,**f**) Knocking down of indicated molecules in basal cells reduced colony (**e**) and sphere (**f**) formation. (**g**) Loss of indicated signalling in basal cells promotes differentiation. qRT–PCR analysis of *AR*, *KLK3* and *KRT18* in spheres treated with SU5402 (5 μM), DAPT (10 μM), XAV-939 (20 μM) or DMSO in the presence of dihydrotestosterone (DHT). Experiments for the first two and last inhibitors were performed in HPCa207N and HPCa208N basal cells, respectively. The *P* value was calculated using Student's *t*-test *P<0.05 and **P<0.01. Data represent means±s.d. from a representative experiment of at least 2 biological repeats in different human samples (**c**,**f**,**g**).

GSEA and IPA uncovered important signalling pathways enriched in basal cells including TGF-β, NOTCH, WNT/TCF, IGF, FGF, STAT3/IL6 and others (Supplementary Fig. 1e and 3a–c; and Supplementary Data 2). For instance, immunofluorescence of FGFR3 validated our RNA-Seq data and revealed its expression preferentially in the basal layer (Fig. 3a). We systematically investigated some of these pathways in regulating primary basal stem/progenitor activities. Given that each pathway has a large number of components, we first used the pathway-specific pharmacological inhibitors to interrogate their roles in regulating basal cell activity. For pathways of particular interest, small interfering RNA (siRNA)-mediated knock-down experiments were performed to validate the inhibitor results.

Overall, all pathway inhibitors examined demonstrated dose-dependent inhibitory effects on basal stem/progenitor activities. Surprisingly, basal cells were relatively tolerant of inhibitors of the TGFβR, NOTCH and WNT pathways in 2D cell growth (Fig. 3b) but were very sensitive to these inhibitors in 3D sphere-formation (Fig. 3c) assays. In particular, DAPT, an inhibitor of NOTCH, only slightly affected basal cell proliferation at ≤20 μM (Fig. 3b) but significantly inhibited sphere formation (both number and size) at ≤10 μM (Fig. 3c). This is consistent with recent genetic studies showing that disrupting canonical Notch impairs the differentiation of murine prostate basal SCs but not their proliferation^21^. Similar results were observed in other primary basal cells (Supplementary Fig. 3d). Consistent with the reported requirement of IGF and FGF in establishing a regulatory SC niche in cultured human ESCs^22^, and promotion by STAT3 of stem-like phenotypes in normal prostate and PCa cells^23^,^24^, basal cells exhibited a high sensitivity to inhibitors of FGFR, IGF1R and STAT3 signalling (Fig. 3b–d). Consistently, knocking down *FGFR3*, *NOTCH1* and *CTNNB1* also greatly reduced colony and sphere formation in basal cells (Fig. 3e,f and Supplementary Fig. 3e–g).

A key feature of basal SC properties is reflected in their ability to differentiate into luminal-like cells. As blockade of FGFR3, NOTCH1 and CTNNB1 pathways impaired basal cell proliferation, we reasoned that inhibition of these pathways might also promote cellular differentiation. As shown in Fig. 3g, SU5402-, DAPT- and XAV-939-treated spheres exhibited markedly increased mRNA levels of *AR*, *KLK3* (*PSA*) and *KRT18*, respectively. These data suggest that blocking signalling pathways important for cell proliferation could promote differentiation of prostatic basal stem/progenitor cells.

### Ribosome biogenesis and MYC programme in basal cells

**Figure 4:**
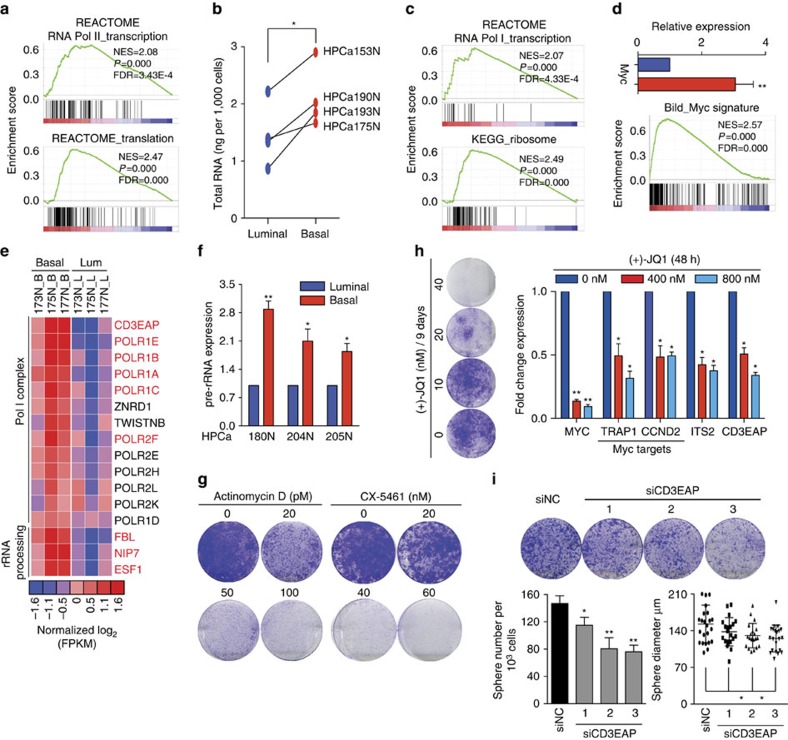
Enhanced rRNA transcription and ribosome biogenesis in basal cells. (**a**–**c**) GSEA showing enrichment of indicated gene signatures in basal cells (**a**,**c**) and basal cells possess higher total RNA contents than luminal cells (**b**). For **b**, cells were purified from the indicated benign prostate tissues, lysed, and total RNA content per 1,000 cells determined. (**d**,**e**) Overexpression of Myc and its transcriptional programme in basal (red) compared to luminal (blue) cells (**d**) and heatmap of relative expression levels of Pol I complex components and key genes involved in rRNA processing in basal and luminal (Lum) cells (**e**). Genes upregulated in basal cells with either FDR<0.05 or *P*<0.05 are coloured in red. See the ‘Methods' section for detail. (**f**) Pre-rRNA expression determined by qRT–PCR of the internally transcribed spacer (ITS2) of the human 47S pre-rRNA in paired fresh basal and luminal cell populations purified from three benign prostate samples. (**g**) Effects of Actinomycin D and CX-5461 on colony formation of HPCa208N basal cells. (**h**) Effects of JQ1 on basal cell proliferation (left) and expression of indicated genes (right). Cells derived from HPCa204N and HPCa207N were used, respectively. (**i**) Knocking down of CD3EAP in HPCa208N basal cells reduces colony formation (upper), sphere formation and sphere size (lower). The *P* value was calculated using Student's *t*-test *P<0.05 and **P<0.01. Data represent means±s.e.m. from 3 biological repeats (**d**), and means±s.d. from a representative experiment of at least 2 biological repeats in human samples (**f**,**h**,**i**).

SCs generally exhibit high levels of global transcription^25^. GSEA showed the enrichment of signatures of Pol II-mediated transcription and protein translation in basal cells (Fig. 4a). In support, freshly purified basal cells contained higher total RNA content than matched luminal cells (Fig. 4b). Protein synthesis inhibitor cycloheximide significantly inhibited basal cell proliferation at as low as 20 nM (Supplementary Fig. 4a). Strikingly, basal cells also preferentially upregulated the signatures of Pol I transcription and ribosome biogenesis (Fig. 4c). Pol I-dependent transcription governs abundance of rRNA and directly regulates cellular translational and thus proliferative capacity. It is well-known that MYC regulates rRNA synthesis and ribosome biogenesis through direct activation of Pol I and transcriptionally increasing the levels of Pol I subunits^26^. In support of this connection, *MYC* was upregulated in basal cells, along with the MYC targets and MYC-dependent transcriptional programme (Fig. 4d and Supplementary Fig. 4b). Moreover, several key Pol I complex subunits (Supplementary Fig. 4c) and genes involved in rRNA processing were also upregulated in basal cells (Fig. 4e). qRT–PCR analysis in three other pairs of matching basal and luminal populations revealed enhanced rRNA transcription rate in basal cells (Fig. 4f and Supplementary Fig. 4d,e).

Several recent reports have linked rRNA transcription and ribosome biogenesis to SC activities^27^,^28^,^29^. To examine the role of rRNA transcription in basal stem/progenitor cell activities, we utilized Actinomycin D, a transcriptional inhibitor that mainly inhibits rRNA transcription when used at a low concentration^27^. We observed that Actinomycin D, at as low as 20 pM, greatly inhibited cell proliferation (Fig. 4g). We also treated freshly purified basal cells with CX-5461, which selectively inhibits Pol I-dependent transcription^30^, and observed that CX-5461 completely blocked the cell proliferation at 40 nM (Fig. 4g and Supplementary Fig. 4f). Notably, both inhibitors also severely impaired the sphere-formation ability (Supplementary Fig. 4g).

Next, we utilized JQ1 to investigate the role of MYC transcriptional programme. As a selective small-molecule inhibitor of BET bromodomains, JQ1 transcriptionally downregulates *Myc* itself and its target genes^31^. Primary basal cells were exquisitely sensitive to JQ1, which significantly attenuated MYC transcriptional programme by decreasing *MYC* itself and its targets (Fig. 4h). Perturbation of MYC programme also decreased the rate of pre-rRNA transcription (ITS2) and the levels of key Pol I subunit *CD3EAP* (Fig. 4h). These results suggest that MYC positively regulates basal cell proliferation, at least partially, through enhancing Pol I-mediated rRNA transcription. In support, siRNA-mediated knock down of the key subunit of Pol I complex *CD3EAP* inhibited cell proliferation and sphere formation, as well as the sphere sizes (Fig. 4i). Collectively, these data reveal enhanced Pol I transcription and ribosome biogenesis in prostate basal epithelial cells, which is required for their stem/progenitor activities and regulated by the MYC transcriptional programme.

### Intrinsic proneural properties of prostatic basal cells

Surprisingly, our RNA-Seq data, for the first time, revealed a large number of ‘proneural' genes in both basal and luminal cells, although, interestingly, the two cell populations preferentially expressed rather different proneural gene sets (Supplementary Data 1). Basal cells overexpressed many more proneural genes than luminal cells (11.14 versus 7.55%), many of which are normally associated with neural development, neurogenesis and axonal guidance. In contrast, luminal cells expressed many genes associated with neural signal response and processing. Annotation of these two proneural gene sets (Supplementary Data 1, category of neural and neuronal development) by DAVID and GSEA showed that GO terms related to neural development and neurogenesis were markedly enriched in basal cells whereas luminal cells were enriched in terms/signatures associated with neural sensory perception and response (Supplementary Fig. 5a,b).

**Figure 5:**
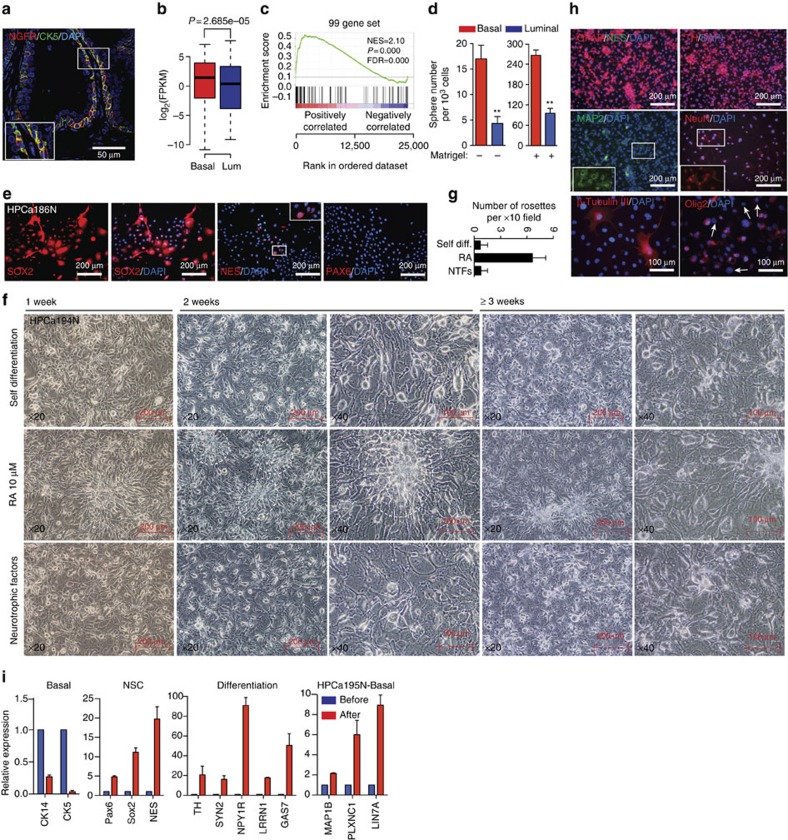
Intrinsic proneural properties of human prostate basal epithelial cells. (**a**) Immunofluorescence of NGFR and CK5 in benign prostate tissue showing the basal localization for NGFR. (**b**,**c**) Gene-expression plot (**b**) and GSEA (**c**) indicate that basal cells overexpress a set of proneural genes (*n*=99, Supplementary Table 4) essential for neural and neuronal development. (**d**) In neurosphere suspension culture condition, primary HPCa202N basal cells generated spheres more efficiently than luminal cells. Values are mean±s.d. (**e**) Immunofluorescence analysis of SOX2, NES (Nestin) and PAX6 in HPCa186N primary basal cell cultures. (**f**) Representative images showing cell morphological changes of basal cells in various culture conditions (See the ‘Methods' section) at different time points after confluence. The white elliptical lines indicate the rosette cluster-like structures. (**g**) Quantification of rosette-like structures in **f**. NTFs, NFs. (**h**) Immunofluorescence analysis of neural lineage markers indicated in end point basal cell cultures shown in **f**. White arrows indicate the cells negative for Olig2 staining. (**i**) qRT–PCR analysis of basal cell and NSC markers and a panel of neural/neuronal genes in basal cells before and after proneural differentiation. The *P* value was calculated using Student's *t*-test *P<0.05 and **P<0.01. Data represent means±s.d. from a representative experiment of at least 2 biological repeats in different human samples (**d**,**g**,**i**).

Immunofluorescence analysis of NGFR revealed a basal localization (Fig. 5a), validating RNA-Seq data. Gene-expression plot (Fig. 5b) and GSEA (Fig. 5c) highlighted an independent signature of 99 essential neural/neuronal development genes (Supplementary Table 2) enriched in basal cells. Considering the demonstrated SC properties of basal cells (Fig. 2), we reasoned that the enriched proneural gene profile might confer on them certain intrinsic traits of neural SCs (NSCs). We performed a classical NSC assay to show that freshly purified basal cells displayed approximately five times higher efficiency in generating ‘neurospheres' in neural culture conditions^32^ with or without Matrigel (Fig. 5d). Strikingly, primary basal cell cultures showed homogeneous and strong expression of SOX2 and sporadic expression of NES, whereas PAX6 was not detectable (Fig. 5e), consistent with its low mRNA level (FPKM<0.1). A recent study reported that SOX2 alone could reprogramme human fibroblasts into multipotent NSCs^33^. Therefore, high levels of SOX2 may provide a mechanism to confer basal cells NSC-like properties. Also, basal cells highly overexpressed *HMGA2* (FPKM=8.62±3.43 in basal versus 0.42±0.2 in luminal), an epigenetic factor that can facilitate the reprogramming of human adult somatic cells into NSCs through an interaction with SOX2 (ref. 34).

During the initial culture of freshly purified basal cells, virtually all cells presented typical flat epithelial morphology. However, upon reaching confluence, we frequently observed clusters of cells resembling classical neural rosettes (Supplementary Fig. 5c, circles). In particular, in post-confluent cultures, numerous cells manifested as morphologically neural-like cells, which were not neuroendocrine cells as they were negative for neuroendocrine markers such as SYN (Supplementary Fig. 5c). Longitudinal tracking and quantification of the morphologically neural-like cells at different culture stages indicated that these neural-like cells were differentiated from basal epithelial cells (Supplementary Fig. 5d,e). Considering the high levels of *SOX2* and *HMGA2*, these results suggest the presence of NSC-like cells in our primary basal cell cultures, which have the potential to differentiate into neural-like cells.

We next investigated the proneural differentiation potential of primary basal cells using three different protocols (see ‘Methods' section). In the default ‘spontaneous' protocol, basal cells were continuously cultured for 3 weeks post confluence without medium change. In the second protocol, 10 μM retinoic acid (RA) was introduced, as it is widely used to induce neural differentiation of pluripotent SCs. In the third protocol, neurotrophic factors (NFs; namely BDNF, GDNF and NGF-β, all at 10 ng ml^−1^) and db-cAMP (0.5 mM) were added to the medium^35^, as basal cells upregulated several receptors (for example, NGFR, NPBWR1). Remarkably, all three protocols resulted in similar neural-like cultures during the time course investigated (Fig. 5f and Supplementary Fig. 5e), although RA treatment induced more rosette-like structures (Fig. 5g). Characterization of the end point cultures revealed strong staining of astrocyte marker GFAP and neuronal marker TH, and relatively weak staining of mature neuronal markers MAP2, NeuN and β-tubulin III (Fig. 5h). The majority of the cells were also positive for oligodendrocyte precursor marker Olig2 (Fig. 5h). These results, together, suggest that the primary prostatic basal cells have the capacity to differentiate into neural progenitor-like cells, evidenced by expression of multiple progenitor but not mature neuronal markers. The specificity of antibodies used was validated by immunofluorescence analysis of mouse brain tissues (Supplementary Fig. 5f). qRT–PCR analysis showed that, upon induction of differentiation in the default protocol, basal cells attenuated their basal epithelial identity and upregulated the levels of NSC markers and a panel of neural/neuronal genes (Fig. 5i and Supplementary Fig. 5g). Finally, single-cell clonal analysis confirmed the ability of basal cells to differentiate into neural-like cells (Supplementary Fig. 5h). Collectively, these results suggest that prostatic basal cells intrinsically express a neurogenic gene profile and are endowed with the ability to differentiate along neural lineages.

The prostate is an organ richly innervated by the autonomic nervous system, and autonomic nerve development contributes to PCa progression^36^. Considering that nerves are a common feature of the microenvironment, we speculated that the presence of neurons, together with other types of neural cells, in the stroma might contribute to the proneural properties of basal epithelial cells. Indeed, immunofluorescence analysis revealed rare TH^+^, NES^+^ and β-Tubulin III^+^ nerve fibres and abundant GFAP^+^ cells proximal to the basal layer (Supplementary Fig. 6a), suggesting that basal epithelial cells may also respond to and are regulated by neural/neuronal signals from the underlying microenvironment. In support, a cocktail of NFs greatly promoted migratory and invasive capabilities of primary basal cells (Supplementary Fig. 6b). These data suggest that the stromal microenvironment might contribute to and regulate the neurogenic property of basal cells.

### Proneural genes regulate basal stem/progenitor activities

We have, for the first time, identified a proneural gene-expression profile in basal cells, but the functional significance for these genes in regulating the basal cell properties remains unknown. Among them, several, that is, HMGA2, CDH13, NGFR and NRG1 (Fig. 1i) are of particular interest. HMGA2 is highly expressed in and also regulates the murine fetal NSCs^37^. CDH13 is a GPI-anchored member of cadherin superfamily with regulatory functions in axon growth during neural differentiation, and does not function through cell–cell adhesion due to the lack of a cytoplasmic domain characteristic of other cadherins. Interestingly, CDH13 is generally lost in cancer, including PCa, due to promoter hypermethylation^38^. NGFR has been previously reported as a prostate basal cell marker underexpressed in PCa^39^. NRG1 is a glycoprotein that interacts with and activates ERBB receptors. Notably, *ERBB1* (*EGFR,* FC=2.15, FDR=0.054; Supplementary Fig. 1f) and *ERBB3* (FC=3.62, FDR=0.0009) are significantly overexpressed in basal and luminal cells, respectively, suggesting the possible involvement of NRG1-mediated signalling in regulating both cell layers. Consistent with this possibility, basal mammary epithelial cells control luminal progenitor maturation and function through a paracrine p63-NRG1 axis during lactogenesis^40^.

**Figure 6:**
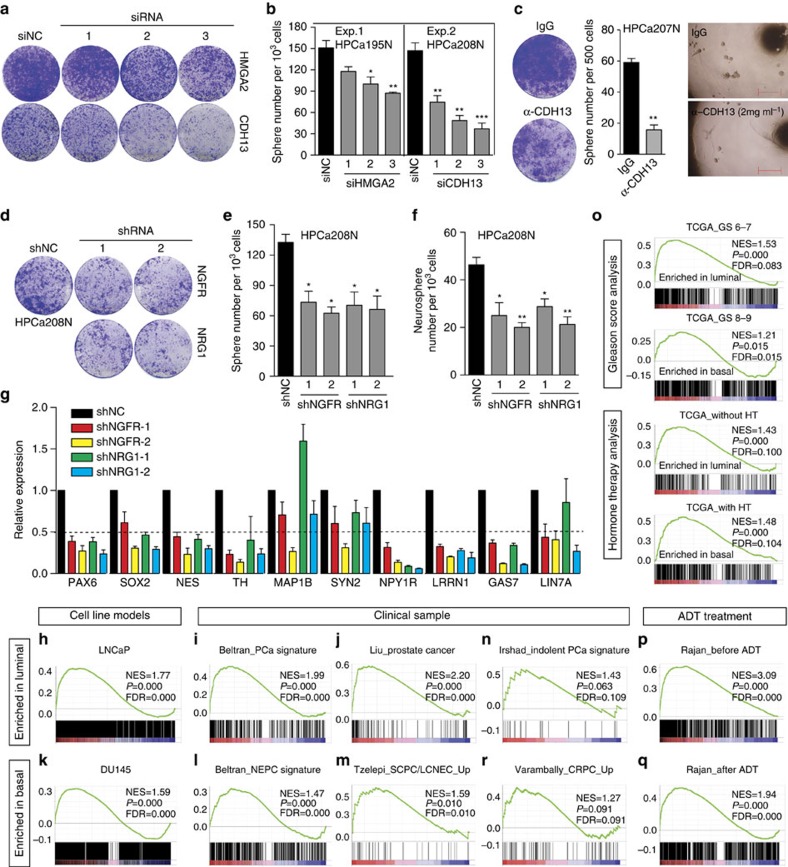
Proneural genes regulate prostatic basal cell stem/progenitor activities and the basal gene-expression profile is linked to aggressive PCa. (**a**,**b**) Knocking down of *HMGA2* and *CDH13* reduce 2D colony (**a**) and 3D sphere (**b**) formation in primary basal cells. (**c**) Neutralization of CDH13 protein by blocking antibody inhibits basal cell proliferation and sphere-forming ability. (**d**,**e**) Knocking down of *NGFR* and *NRG1* by shRNA in basal cells reduces colony (**d**) and sphere (**e**) formation. (**f**) Knocking down of *NGFR* and *NRG1* by shRNA inhibits neurosphere formation in primary basal cells. Bars in **e** and **f** represent the mean±s.d. (**g**) qRT–PCR analysis of NSC markers and a panel of neural/neuronal genes in HPCa208N basal cells treated with shRNAs after proneural differentiation. (**h**–**r**) GSEA showing enrichment of indicated PCa gene signatures in human benign prostatic luminal and basal cells. See the ‘Methods' section for details. The *P* value was calculated using Student's *t*-test *P<0.05, **P<0.01, ***P<0.001. Data represent means±s.d. from a representative experiment of at least 2 biological repeats in different human samples (**b**,**c**,**e**,**f**,**g**).

Knocking down of *HMGA2* and *CDH13* by siRNA significantly impaired basal cell proliferation and stemness (Fig. 6a,b). Experiments using blocking antibody further confirmed the effect of CDH13 knock down on basal cell biology (Fig. 6c). Although the knock-down efficiency of *HMGA2* siRNAs was relatively low, the results could be readily reproduced in primary basal cells from other benign prostate tissues (Supplementary Fig. 7a,b). Likewise, lentiviral shRNA-mediated knock down of *NGFR* and *NRG1* inhibited basal cell colony and sphere formation (Fig. 6d,e and Supplementary Fig. 7c), neurosphere formation (Fig. 6f), as well as the proneural differentiation (Fig. 6g). These data suggest that the proneural genes are functionally important in regulating basal stem/progenitor activities, as well as their capacity to undergo proneural differentiation.

### Basal cell gene profile is linked to aggressive PCa

We determined whether our global transcriptomic profiles of normal basal and luminal epithelial lineages could be linked to clinical features of PCa by comparing with multiple clinical sample (including TCGA) and cell line data sets. It is well-known that the majority of untreated primary PCa present as adenocarcinomas while a small subset (1–5%) of patient tumours is classified as undifferentiated or anaplastic PCa variants frequently termed as small cell PCa or neuroendocrine PCa. These tumours have a clinically aggressive behaviour, lack AR expression, and are refractory to androgen-deprivation therapy (ADT). Significantly, such aggressive variants markedly increase in castration-resistant PCa (CRPC) patients^41^. GSEA showed that typical clinical PCa and LNCaP cells presented a luminal cell-like gene-expression profile (Fig. 6h–j). In contrast, the basal cells were greatly enriched in gene signatures associated with aggressive PCa including neuroendocrine PCa^42^, small cell PCa/LCNEC (large-cell neuroendocrine carcinoma)^43^, and PC3 and Du145 (Fig. 6k–m and Supplementary Fig.7d), suggesting a global basal cell-like gene-expression profile for these PCa variants. Importantly, a 19-gene indolent PCa signature^44^ was enriched in luminal cells (Fig. 6n), suggesting a predictive value of luminal cell gene profile in distinguishing indolent versus aggressive disease. In support, when we performed clustering analysis of TCGA-PCa data, we extracted two gene signatures corresponding to patients with low and high Gleason score (GS; Supplementary Fig.7e), and observed a strong enrichment of low GS signature in luminal cells and of high GS signature in basal cells (Fig. 6o).

Two RNA-Seq data sets generated from PCa patients before and after ADT^45^ were utilized to investigate whether our transcriptomes could distinguish CRPC versus treatment naive PCa. Strikingly, the ‘before-ADT' gene-expression profile resembled that of luminal cells (Fig. 6p) whereas CRPC after ADT expressed a basal-like profile (Fig. 6q). A similar association between basal gene profile and CRPC was observed in two Oncomine data sets (Fig. 6r and Supplementary Fig. 7f). Finally, in TCGA-PCa patients, the gene signature in patients with or without hormonal therapy (HT) was greatly enriched in our benign basal and luminal cells, respectively (Fig. 6o and Supplementary Fig. 7g). Compared with the signature of untreated high GS patients, the signature of high GS patients after HT became further enriched in basal cells evidenced by increased NES (1.21 versus 1.48; Fig. 6o), suggesting that HT induced a further shift of gene expression towards a basal-like profile. Finally, Oncomine concept analysis showed that 10 basal and 18 luminal proneural genes were up and downregulated, respectively, in metastatic versus primary PCa (Supplementary Fig. 7h), suggesting that metastatic PCa are more likely to express a basal-like profile. Further analysis showed that the expression levels of some basal (for example, *GLS*, *NME1*) and luminal (for example, *DLGAP1*, *PTPRN2*) proneural genes were, respectively, associated with poor and better patient survival (Supplementary Fig. 7i).

## Discussion

**Figure 7:**
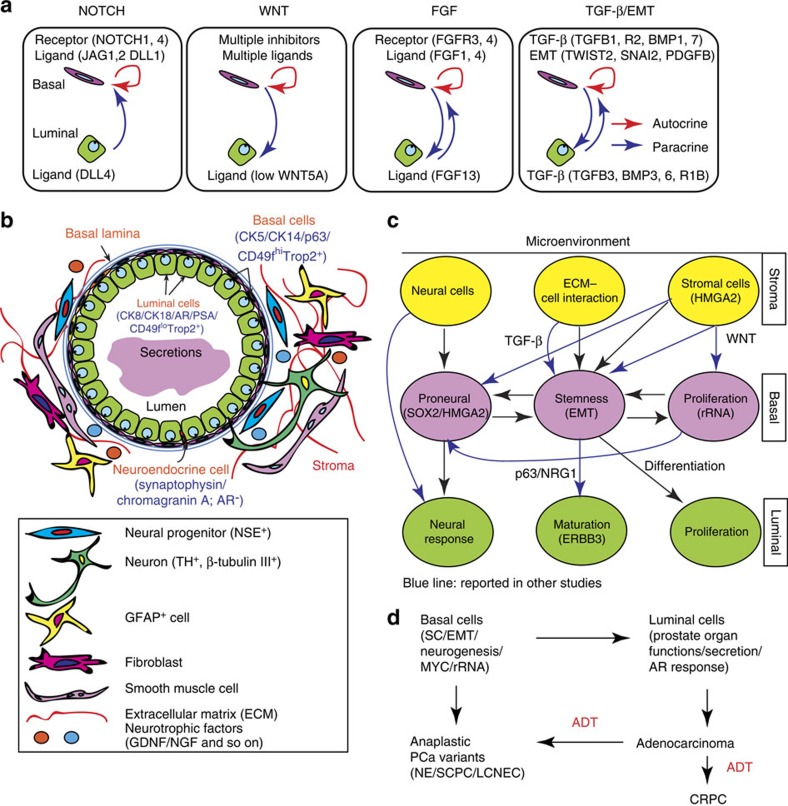
Transcriptome-derived models of crosstalk between prostatic epithelial lineages and between epithelial cells and microenvironment. (**a**) Schematic illustration of crosstalk in representative signalling pathways between prostatic basal and luminal cells. Preferentially expressed genes in each lineage are indicated. (**b**) A schematic illustrating potential crosstalk between epithelial cells and ECM and stromal cells. (**c**) Schematic illustration of reciprocal signalling crosstalk between basal, luminal cells and stromal compartments. Black arrows indicate data obtained in this study and blue arrows the interactions reported in the literature. (**d**) Basal cells could potentially function directly as the cells-of-origin for anaplastic variant PCa and/or indirectly as the cells-of-origin for adenocarcinomas via differentiation into luminal cells.

The current study has made the following significant findings (see Supplementary Discussion). First, our study uncovers unique SC- and EMT-enriched gene-expression profile in unperturbed basal cells that support the long-held hypothesis that the human prostate basal cell layer harbours primitive SCs. Second, we report the surprising finding that basal cells are enriched in genes normally associated with neurogenesis. In contrast, luminal cells preferentially express proneural genes involved in neural signal response and processing. Consistently, primary basal cells can spontaneously or be induced to undergo ‘neural' development *in vitro*, generating NSC-like cells. Combined with the SC features, these transcriptional programs provide a molecular understanding for the reported basal cell plasticity^20^. Third, basal cells express high levels of Pol I-associated rRNA biogenesis genes regulated, at least in part, by the MYC transcriptional programme. MYC is often found overexpressed in PCa, especially metastatic PCa^46^. Increased transcription of rRNA genes by Pol I is a common feature of human cancer. Thus, our data may suggest a rationale for treating anaplastic PCa and CRPC with Pol I inhibition^30^,^47^, as well as targeting MYC and the MYC-mediated transcriptional programme as a therapy for PCa. Fourth, our deep RNA-Seq data provide a rich resource for epithelial lineage-specific genes and markers in the human prostate. Fifth, distinct transcriptomes in basal and luminal cells also suggest cross communications between the two epithelial cell types, as well as between the epithelial compartment and the underlying stroma (Fig. 7; Supplementary Discussion). Understanding such crosstalk will be instrumental for understanding the normal development and tumourigenesis of prostate. Although many of the signalling pathways mentioned in this study are poorly investigated in normal prostate epithelial biology, their functional involvement in PCa development and progression has been widely documented^3^. Last, the basal cell gene-expression profile is linked to adverse clinical features of PCa, indicating a ‘biomarker' value of basal cell gene signature for aggressive PCa. Importantly, the molecular resemblance of basal cells to anaplastic PCa and CRPC provides a common molecular understanding of these diverse and poorly characterized aggressive PCa subtypes and implicates basal cells as the cell-of-origin for these variant PCa (Fig. 7d). It should be noted that while this manuscript was under review, another paper reported similar findings in linking the basal cell gene expression to aggressive PCa^48^.

Overall, by detailed transcriptome analysis of unperturbed human benign prostatic basal and luminal cells, we uncover many intrinsic molecular and functional differences in the two cell types that are linked to their distinct biological properties. Further characterizing these differences will shed fresh lights on the aetiology of and developing novel therapies against both adenocarcinomas and variant PCa.

## Methods

### Human primary prostate tissue processing and FACS

All primary human PCa (HPCa; Supplementary Table 1) patient benign samples were obtained with the written informed consent from the patients in accordance with federal and institutional guidelines and with the approved IRB protocols (MDACC LAB04-0498). HPCa processing protocol was previously described^49^. The final dissociated single-cell suspension was stained with PE-CD49f (1:200, Clone GoH3, Biolegend, San Diego, CA), APC-Cy7-CD45 (1:100, Clone HI30, eBioscience, San Diego, CA) and APC-Trop2 (1:50, Clone 77220, R&D Systems, Minneapolis, MN) antibodies. FACS analysis and sorting were performed by using the BD Aria or Fusion (BD Biosciences, San Jose, CA). Propidium iodide was added before FACS analysis to separate viable from dead cells. In this study, the CD45^+^ immune cells were excluded, and Trop2^+^ epithelial cells were collected according to high (basal-enriched) or low (luminal-enriched) expression of CD49f.

### Deep RNA-Seq and data processing

The FACS-purified human prostate basal (Trop2^+^CD49f^hi^) and luminal (Trop2^+^CD49f^lo^) epithelial populations were subject to total RNA extraction by RNeasy mini kit (Qiagen, Valencia, CA). cDNA libraries were constructed by using TruSeq Stranded Total RNA Preparation Kit (Illumina, cat #: RS-122-2301), which contained Ribo-Zero Gold and allowed the depletion of rRNA. Importantly, we only amplified our libraries with 10 PCR cycles (instead of 15 cycles suggested by the manufacturer) to minimize amplification-induced noise. Purified libraries were quantified using a Kapa library quantification kit (KAPA Biosystems, Wilmington, MA), and then loaded on cBot (Illumina, San Diego, CA) at a final concentration of 10 pM to perform cluster generation, followed by 2 × 76 bp sequencing on HiSeq 2000 (Illumina, San Diego, CA). Two libraries were pooled and loaded on HiSeq 2000, producing an average of 400 million 76-mer reads per lane. From each sample, we obtained about 100 million pairs of reads (200 M reads), indicating the high depth of sequencing. We mapped the sequencing reads to the reference human genome sequence (NCBI 36.1 [hg19] assembly) using TopHat v2.0.9 (ref. 50) and Bowtie v2.1.0 (ref. 51). Then, we assembled the alignments into gene transcripts and calculated their relative abundance using Cufflinks v2.1.1 and HTSeq v0.5.3p9 (ref. 52). DESeq v1.10.1 (ref. 53) was used as a statistical procedure to call DEGs in different samples. For quality check, MA plot was generated using log ratios and average expression.

### DEG calling and novel markers for each cell lineage

In RNA-Seq analysis, the *q* value is an adjusted *P* value, taking into account the FDR. A *P* value of 0.05 indicates that 5% of all tests will be false positives. An FDR-adjusted *P* value of 0.05 implies that 5% of the tests found to be statistically significant (for example, by *P* value) will be false positives. Therefore, FDR has a greater power than *P* value, and we have mainly relied on FDR to gauge DEGs. To define DEGs, we used very stringent statistic threshold of ≥2 FC and FDR <0.05 to generate manageable lists in order for us to perform manual curation to classify each DEG in each cell type into non-redundant functional categories. Using the above statistical threshold, we identified a consensus of 853 DEGs upregulated in basal and 940 DEGs in luminal cells (Supplementary Data 1). Notably, to avoid the misunderstanding that genes not presented in the ‘stringent' lists are not DEGs, we also listed genes that passed a relatively loose but still statistically significant cutoff (that is, FC≥2 and *P*<0.05) in Supplementary Data 1. This latter cutoff resulted in more DEGs in basal (*n*=1,432) and luminal (*n*=1,548) cell populations (Supplementary Data 1). For example, FGFR3 (Fig. 3a) and some Pol I complex subunits (Fig. 4e; for example, POLR1B (*P*=0.006, FDR=0.069), POLR1C (*P*=0.006, FDR=0.069), NIP7 (*P*=0.005, FDR=0.060), and ESF1 (*P*=0.006, FDR=0.063) were not in the list with FDR<0.05, but were in the list with *P*<0.05. For Fig. 3a, the reason we chose FGFR3 (*P*=0.006, FDR=0.07) for demonstration was its abundance over other differentially expressed FGFRs (for example, the mean FPKM in basal cell, FGFR3=11 versus FGFR4=1), although its FDR was slightly above the stringent cutoff of 0.05. To get more reliable and manageable results, we mainly used the fewer DEGs lists for bioinformatics analysis.

For Fig. 1i, we identified the top 50 putative marker genes specific for each lineage inferred from transcriptomes based on both relative differential expression (FC) and absolute expression levels (normalized read counts). To increase the confidence of this selection, we scanned the genes from the stringent DEGs lists. Thus, the genes showing high-RNA expression (normalized read counts>300) in both cell types, regardless of the differential FC, would be excluded due to the high probability of protein expression in both cell types. Likewise, genes showing high FC difference between the two cell types but having minimal RNA expression in either cell type (that is, normalized read counts<300, indicating the less probability of robust protein expression) would also be eliminated. Note that normalized read counts of 300 (quite high) is an arbitrary set-up to increase the reliability of this selection. Using these criteria, we could identify >100 genes unique for each cell type, and the top 50 were shown in Fig. 1i. Notably, FGFR3 is not in the top 50, but we included it in Fig. 1i owing to the experimental data and for the reasons discussed above.

### IPA and GSEA

For GO analysis, IPA (Qiagen, Valencia, CA) and DAVID version 6.7 (ref. 54) were used with gene symbols. GSEA was carried out by using the curated gene sets (C2) of the Molecular Signature Database (MSigDB) version 4.0 provided by the Broad Institute (http://www.broad.mit.edu/gsea/)^55^. Note that to dissect the profile of each cell type, the list of DEGs and entire detectable genes derived from each sample were used for IPA and GSEA, respectively. In particular, we followed the standard procedure as described by GSEA user guide (http://www.broadinstitute.org/gsea/doc/GSEAUserGuideFrame.html). The FDR for GSEA is the estimated probability that a gene set with a given NES (normalized enrichment score) represents a false-positive finding, and an FDR<0.25 is considered to be statistically significant for GSEA.

### Generation of signatures from PCa cell lines and literature

The majority of the gene signatures used in this study were obtained from MSigDB, unless noted in the main text or here. To compare our epithelial cell transcriptomic data with gene-expression profiles of PCa cell lines, we have taken two complementary approaches to perform GSEA. First, the RNA-Seq data of several PCa cell lines (that is, LNCaP, Du145 and PC3) have been recently generated by our colleagues (Drs M Estecio and C Liu). We used our well-defined basal and luminal signatures to perform GSEA against the three PCa cell RNA-Seq data, finding enrichment of luminal signature in LNCaP cells (data not shown), and of basal signature in PC3 (Supplementary Fig. 7d) and Du145 (data not shown) cells. Alternatively, we extracted the cell line signatures and then performed GSEA against our basal and luminal RNA-Seq data. For example, the LNCaP signature (Fig. 6h) comprised genes only expressed by LNCaP plus the genes overexpressed in LNCaP compared with PC3, whereas the Du145 (Fig. 6k) and PC3 (data not shown) signatures were composed of genes only expressed by them plus the overexpressed genes compared with LNCaP. As expected, the two methods generated highly concordant results. For data sets or signatures from published literature, we collected them from corresponding Supplementary Information. These references and brief details of these studies were summarized in Supplementary Table 3.

### Analysis of TCGA-prostate adenocarcinoma RNA-Seq data

Currently, the TCGA-PCa project contains a total of 498 cases, in which 497 cases have mRNA (RNA-Seq) data. On the basis of the availability of the matched RNA-Seq and clinical data (for example, GS and treatment information), we found 487 cases useful for our analysis. According to the GS distribution, 45 cases are GS6, 246 GS7, 62 GS8, 131 GS9 and 3 GS10. We assigned the 3 GS10 patients into the group of GS9+10. In Supplementary Fig. 7e, to create gene signatures specific to low (*n*=605 genes) and high (*n*=639 genes) GS patients, respectively, we used all detectable genes (*n*=20,502) and objectively applied them to the shrunken centroid supervised algorithm (PAMR)^56^ to perform feature selection, resulting in 1,244 genes that were associated with GS based on the prediction error. Interestingly, among the 487 informative cases, 64 patients were treated with HT, providing us a unique source to investigate the HT-induced changes in global gene-expression profiles. In this treatment group, 5 are GS7, 14 GS8 and 45 GS9. Therefore, to generate signatures tightly associated with treatment (*n*=435 genes) versus no treatment (*n*=222 genes), and considering the majority of treated cases were GS≥8, we only included GS8 and GS9 patients (*n*=59) for analysis, and used them to compare the rest of patients (untreated *n*=137) with GS≥8. Again, based on the TCGA-PCa RNA-Seq data, we applied multiple *t*-tests, and selected genes whose expression values were >2.4 and testing raw *P* values <0.05. In total, we observed 657 genes that could discriminate the treatment versus non-treatment groups, in which 435 genes (signature with HT) were upregulated in the treatment group, whereas 222 genes (signature without HT) were upregulated in non-treatment group (Supplementary Fig. 7g).

### Human primary prostate cell cultures

Either the bulk dissociated prostate epithelial cells or the FACS-purified basal and luminal cell populations were plated in T25 flasks precoated with PureCol (Advanced BioMatrix, San Diego, CA). We mainly used WIT medium (Stemgent, Cambridge, MA, cat no# 00-0045-500) supplemented with 10 μM of p160 ROCK inhibitor Y-27632 dihydrochloride (Selleckchem, Houston, TX) in this study. WIT Medium is a serum-free defined medium originally optimized for the robust culture of human primary mammary epithelial cells without the need of feeder cells^57^. PrEGM (Prostate Epithelial Cell Growth Medium; Lonza, Walkersville, MD) has been widely used in culturing prostate cells in the field^58^; however, we chose the WIT medium for most of our studies because we have observed that the WIT medium supports human primary prostate cells better than PrEGM. For cell passaging, the Trypsin-EDTA for Primary Cells (ATCC PCS-999-003) and Trypsin Neutralizing Solution (ATCC PCS-999-004) were utilized. In this study, freshly purified primary basal cells and short-term expanded cultures (<passage 3) were utilized in a spectrum of *in vitro* and *in vivo* assays to characterize the epithelial biology.

### Colony-formation and sphere-related assays

For colony-formation assays^59^, we plated primary prostatic cells at a low density (that is, 800–1,000 cells per well) in a precoated six-well dish, and let cells grow for 7–9 days before the visualization of the culture by crystal violet staining. For inhibitor studies, we usually plated 1,000 cells per well in normal medium at day 1, and then added the inhibitors at varying concentrations on day 2. For sphere-formation assays^60^, cells were suspended in 1:1 Matrigel (BD Biosciences, San Jose, CA)/WIT in a total volume of 100 μl. The mixtures were then plated around the rim of wells in a 12-well plate and allowed to solidify in 37 °C incubator for 25 min, followed by addition of 1 ml of warm WIT medium. Usually 7–9 days after plating, spheres with a diameter over 50 μm were counted. For inhibitor studies in sphere-formation assays, we plated the cells at day 1, and then replaced the medium containing varying concentrations of inhibitors at day 2. For sphere-based differentiation assays, we first established and grew the spheres for 6–7 days in dihydrotestosterone -free medium, and then added the inhibitors and dihydrotestosterone for another 3–5 days of prolonged culture. Dihydrotestosterone has been shown to further induce differentiation of prostate sphere cells^60^. For all above experiments, we ran a minimum of triplicate wells for each condition and repeated experiments in different patient-derived cells whenever feasible.

### Neural sphere-formation assay

The neural sphere formation, a classical NSC assay, was used to measure the proneural or NSC-like properties of prostatic basal cells. Varying numbers of freshly purified human prostatic basal and luminal cells were seeded in serum-free neural media with or without the presence of 5% Matrigel in 96-well ultra-low attachment plates. The neural media^32^ is consisted of DMEM/F12 supplemented with B27, N2, 1 × Glutamax, EGF and Pen/Strep (all from Life Technologies, NY). In some cases (for example, see Fig. 6f), 5% Matrigel was included in the neural media. The number and size of neural spheres formed were generally measured 7–9 days after initial culturing.

### Proneural differentiation protocols

We investigated the proneural differentiation potential of primary basal cells using three different experimental protocols (Fig. 5f). In the default ‘spontaneous' protocol, basal cells were continuously cultured for 3 weeks in WIT post confluence without medium change. In the second protocol, 10 μM RA was introduced, as RA is widely used to induce neural differentiation of pluripotent SCs. In the third protocol, NFs (BDNF, GDNF and NGF-β, all at 10 ng ml^−1^) and db-cAMP (0.5 mM) were added in the medium^35^, as basal cells upregulated several receptors for these NFs (for example, NGFR, NPBWR1). To phenotypically characterize the end point cultures, we stained the cells with antibodies against well-known neural/neuronal lineage markers (that is, GFAP, TH, MAP2, SOX2, NES, RBFOX3 (also known as NeuN), β-Tubulin III, and OLIG2). At molecular level, a panel of neural/neuronal related genes were analysed by qRT–PCR to reveal their expression changes along the proneural differentiation.

### Histology and immunofluorescence staining

Hematoxylin and eosin and immunofluorescence staining was performed on either 5-μm paraffin-embedded or OCT-frozen sections. Basic immunofluorescence procedures have been described previously^59^. For staining of cell cultures, cells were first grown on glass coverslips precoated with PureCol (Type I collagen; Advanced BioMatrix, San Diego, CA), then fixed with 4% paraformaldehyde containing 5% sucrose (pH 7.2). The coverslips or the tissue slides were blocked with Background Sniper (Biocare Medical, Concord, CA) for 30 min, followed by primary antibody incubation overnight at 4 °C. Primary antibodies and dilutions used are listed in Supplementary Table 4. Slides were then incubated with secondary antibodies (diluted 1:700 in antibody diluent (Dako, Carpinteria, CA)) labelled with Alexa Fluor 488 or 594 (Invitrogen/Molecular Probes, Grand Island, NY). After washing (3 ×) with PBS, sections were counterstained with 4,6-diamidino-2-phenylindole (DAPI; Sigma-Aldrich, St. Louis, MO) and mounted with ProLong Gold Antifade Mountant (Life Technologies, Grand Island, NY). Immunohistochemistry and immunofluorescence images were captured by Olympus IX71 and Zeiss LSM510 META confocal microscope, respectively.

### RNA isolation and quantitative RT–PCR

Total RNA was isolated from cells using the RNeasy mini kit (Qiagen, Valencia, CA). The first-strand cDNA synthesis was achieved by reverse transcription of RNA using random hexamers and SuperScript III Reverse Transcriptase (Invitrogen). Quantitative RT–PCR was performed using the iQ SYBR Green supermix (BioRad, Hercules, CA) on a 7900HT Fast Real-Time PCR System (Applied Biosystems, Foster City, CA). The primers used in this study are listed in Supplementary Table 4. Normally, the housekeeping gene *GAPDH* or *β-actin* was used as internal control for gene-expression normalization. In particular, *B2M* gene was used as a control for rRNA-related qRT–PCR analysis, since its transcription was not affected by perturbation of either Pol I or MYC activities^47^.

### Migration and invasion assays

Cell migration and invasion assays were performed using Boyden chambers (CellBiolabs, San Diego, CA) according to manufacturer's instructions. Briefly, freshly purified basal and luminal cell populations were loaded into the chambers and cultured in media for 2 days, and the results were visualized by PROTOCOL Hema 3 staining kit (Fisher Scientific, Pittsburgh, PA). Images of the membranes were captured by Olympus IX71. Data was quantified based on the cell number counting of at least five × 20 images. To test the response of prostatic basal cells to neural signals (Supplementary Fig. 6b), primary basal cells were incubated in the chambers in media with or without neural growth factors (20 ng ml^−1^ of BDNF/GDNF/NGF-β, 500 μM GABA and 0.5 mM db-cAMP).

### siRNA-mediated knock-down experiments

To knock down the genes of interest, we used the Trilencer-27 Human siRNA system (OriGene, Rockville, MD). For each gene (FGFR3 (ID2261), NOTCH1 (ID4851), HMGA2 (ID8091), CDH13 (ID1012), CTNNB1 (ID1499) and CD3EAP (ID10849), 3 unique 27mer siRNA duplexes were used. When passaging, the primary human prostatic basal cells were plated in 12-well plates at a desired density and transfected with 400 nM siRNA oligonucleotides or non-targeting controls. Due to the limited transfection efficiency in primary cells, we usually transfected the cells twice at 12 and 24 h after plating. Transfection was performed with Lipofectamine RNAi MAX in WIT medium. Knock-down efficiency was determined by qPCR at 48 h post transfection. At 48–72 h after transfection, cells were trypsinized, counted and seeded in 6-well plates for colony-formation and in 12-well plates for sphere-formation assays. The sequences for all siRNAs are listed in Supplementary Table 5.

### Lentiviral shRNA-mediated knock-down experiments

To establish long-term knock-down experiments, the GIPz-shRNA lentiviral vectors targeting NRG1 (Clone ID: V2LHS_84774 and V3LHS_344002) and NGFR (Clone ID: V2LHS_152261 and V2LHS_152259) were purchased from the MDACC ShRNA and ORFeome Core Facility. Two shRNAs were used to target each gene (Supplementary Table 5). Basic lentiviral procedures were previously described^2^. Lentivirus was produced in 293FT packaging cells and titres determined using GFP positivity in 293FT cells. Primary prostate cells were infected, generally, at a multiplicity of infection of 15 and collected for experiments 48–72 h post-infection. Cells were trypsinized, counted and seeded in 6-well plates for colony-formation and in 12-well plates for sphere-formation assays. Knock-down efficiency of shRNAs on target genes was determined by qPCR.

### Reproducibility of experiments

No statistical method was used to predetermine sample size for all experiments. Since joining the group, D.Z. has worked on >75 HPCa samples. For immunofluorescence staining, including Figs 1i, 2g,f, 3a, 5a,e,h and Supplementary Figs 1h, 2a,b,d, 5c,f,h, 6a,b, at least 2 different HPCa samples were used, and multiple fields were imaged on each slide. For immunohistochemistry analysis (for example, Supplementary Fig. 1f,g), multiple images were taken from different fields on each slide, and 1 or 2 HPCa samples were utilized. For migration and invasion assays (for example, Fig. 2h and Supplementary Figs 2e and 6b), 2 technical replicates were included for each cell type, and experiments were repeated in 2–3 different biological samples. In particular, 5–6 random high magnification (× 20) images were captured for each membrane and used for quantifications of cell numbers. For all qPCR analysis, three technical replicates were included for each sample, and data shown for qPCR analysis was from one experiment that was representative of more than equal to two independent experiments. In addition to repeat experiments using different biological samples when feasible, the drug treatment (for example, inhibitors), siRNA and shRNA-mediated knock-down, and many other experiments were generally repeated at different passages (time points) in the same patient-derived primary prostate epithelial cells. For all sphere-formation assays, 3–6 technical replicates were included for each sample.

### Statistical analysis

Graphpad Prism software was used to calculate mean and standard deviation. In general, Student's *t*-test was used to calculate the statistical significance between the two groups of data. *P*<0.05 is considered statistically significant.

## Additional information

**Accession codes:** The RNA-seq data have been deposited in GEO database under the accession code GSE67070.

**How to cite this article:** Zhang, D. *et al.* Stem cell and neurogenic gene-expression profiles link prostate basal cells to aggressive prostate cancer. *Nat. Commun.* 7:10798 doi: 10.1038/ncomms10798 (2016).

## Supplementary Material

Supplementary Information Supplementary Figures 1-7, Supplementary Tables 1-5, Supplementary Discussion and Supplementary References.

Supplementary Data 1 Genes upregulated by ≥2 fold and FDR<0.05 in human benign prostatic basal epithelial cells compared to the corresponding luminal cells in the RNA-Seq analysis.

Supplementary Data 2 GSEA pathway analysis indicates the gene signatures enriched in human benign prostatic luminal epithelial cells compared to the corresponding basal cells.
