# Supplementary material for: Stem cell and neurogenic gene-expression profiles link prostate basal cells to aggressive prostate cancer
Source: Nat Commun. 2016 Feb 29;7:10798. doi: 10.1038/ncomms10798 (PMC4773505; doi:10.1038/ncomms10798)
Supplement: Supplementary Information — Supplementary Figures 1-7, Supplementary Tables 1-5, Supplementary Discussion and Supplementary References. [file ncomms10798-s1.pdf]

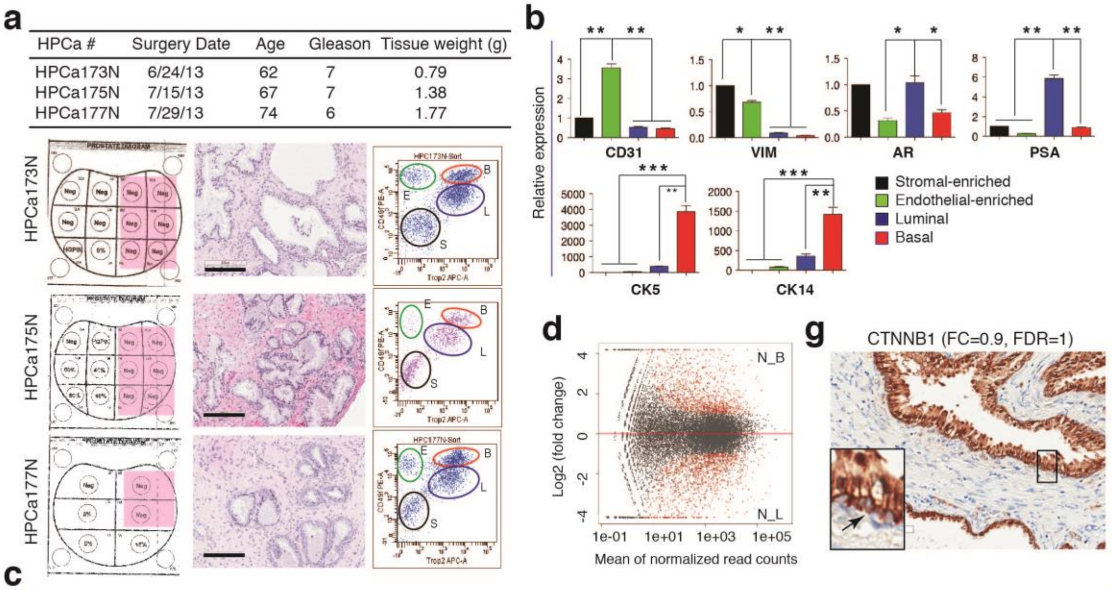


| HPCa # | Surgery Date | Age | Gleason | Tissue weight (g) |
|---|---|---|---|---|
| HPCa173N | 6/24/13 | 62 | 7 | 0.79 |
| HPCa175N | 7/15/13 | 67 | 7 | 1.38 |
| HPCa177N | 7/29/13 | 74 | 6 | 1.77 |

**c**

| Name | frag# | read# | map# | map% | paired_frag# | multi-paired_frag# | multi-paired_frag% | ppaired_frag# | ppaired_frag% |
|---|---|---|---|---|---|---|---|---|---|
| 173N_B | 114,973,410 | 229,946,820 | 209,425,736 | 91.08% | 102,400,114 | 8,085,349 | 7.90% | 101,463,010 | 88.25% |
| 173N_L | 105,204,747 | 210,409,494 | 191,225,565 | 90.88% | 93,504,111 | 10,020,760 | 10.72% | 92,721,593 | 88.13% |
| 175N_B | 111,664,131 | 223,328,262 | 206,290,234 | 92.37% | 101,100,135 | 7,865,301 | 7.78% | 100,070,149 | 89.62% |
| 175N_L | 104,607,699 | 209,215,398 | 193,373,081 | 92.43% | 94,678,578 | 9,060,218 | 9.57% | 93,728,450 | 89.60% |
| 177N_B | 98,300,625 | 196,601,250 | 181,037,375 | 92.08% | 88,683,949 | 7,322,284 | 8.26% | 87,978,753 | 89.50% |
| 177N_L | 99,953,226 | 199,906,452 | 183,550,872 | 91.82% | 89,916,056 | 8,261,177 | 9.19% | 89,276,444 | 89.32% |

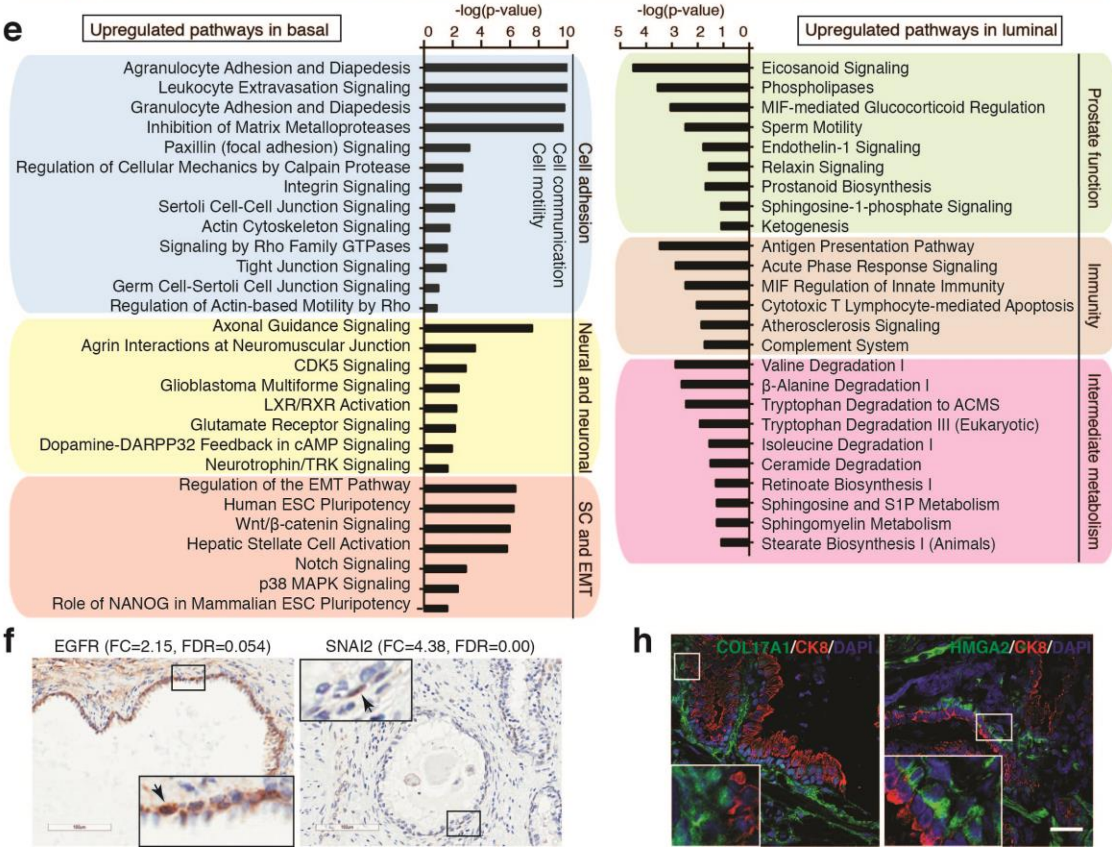

**Supplementary Figure 1.** Human sample information, RNA-Seq parameters, IPA and validation of RNA-Seq data.

**(a)** Basic information of donor samples (HPCa173N, 175N, 177N; also see Supplementary Table 1), the biopsies, representative H&E images, and FACS plots are shown to indicate the benign nature of the prostate tissues used in this study. Scale bars, 200 µm. **(b)** qRT-PCR analysis of transcript levels of lineage markers (CD31 for endothelial cells, VIM for stromal cells, AR and PSA for luminal cells, CK5 and CK14 for basal cells) in distinct purified subpopulations demonstrating successful cell fractionation. The relative transcript abundance was normalized to GAPDH levels. The gene expression in stromal cell-enriched population was arbitrarily set as 1. Results shown (mean ± SD, HPCa153N) were representative data of at least 3 repeat experiments in different patient-derived cell populations. The *P* value was calculated using Student's *t*-test *P < 0.05, ** P< 0.01 and *** P< 0.001. **(c)** Alignment results of RNA-Seq data indicate the high quality of our RNA sequencing data, as evidenced by high mapping rate. multi-paired_frag#, the number of paired fragments that have multiple alignments; ppaired_frag #, the number of properly paired fragments (TopHat alignment software also names it "concordant pair alignment"). **(d)** The MA plot showing the appropriate normalization of our RNA-Seq data. **(e)** IPA of DEGs in human prostate epithelial lineages. The relevant canonical signaling pathways enriched in basal (left) or luminal (right) prostate epithelial cells were grouped respectively into different functional categories and plotted according to their –log(p-value). **(f,g)** IHC analysis of EGFR and SNAI1 (**f**), and CTNNB1 (**g**) in human benign prostate tissues. Consistent with the RNA-Seq data, basal cells preferentially expressed EGFR and SNAI1 compared to luminal cells. In contrast, no differential mRNA and protein levels were found for CTNNB1. The fold change (FC) and FDR values are shown on top of the images. Arrows indicate positive staining in basal cells. Scale bars, 100 µm. **(h)** Double immunostaining of COL17A1 and HMGA2 with CK8 in human benign prostate tissues. Boxed regions are enlarged. Scale bars, 50 µm.

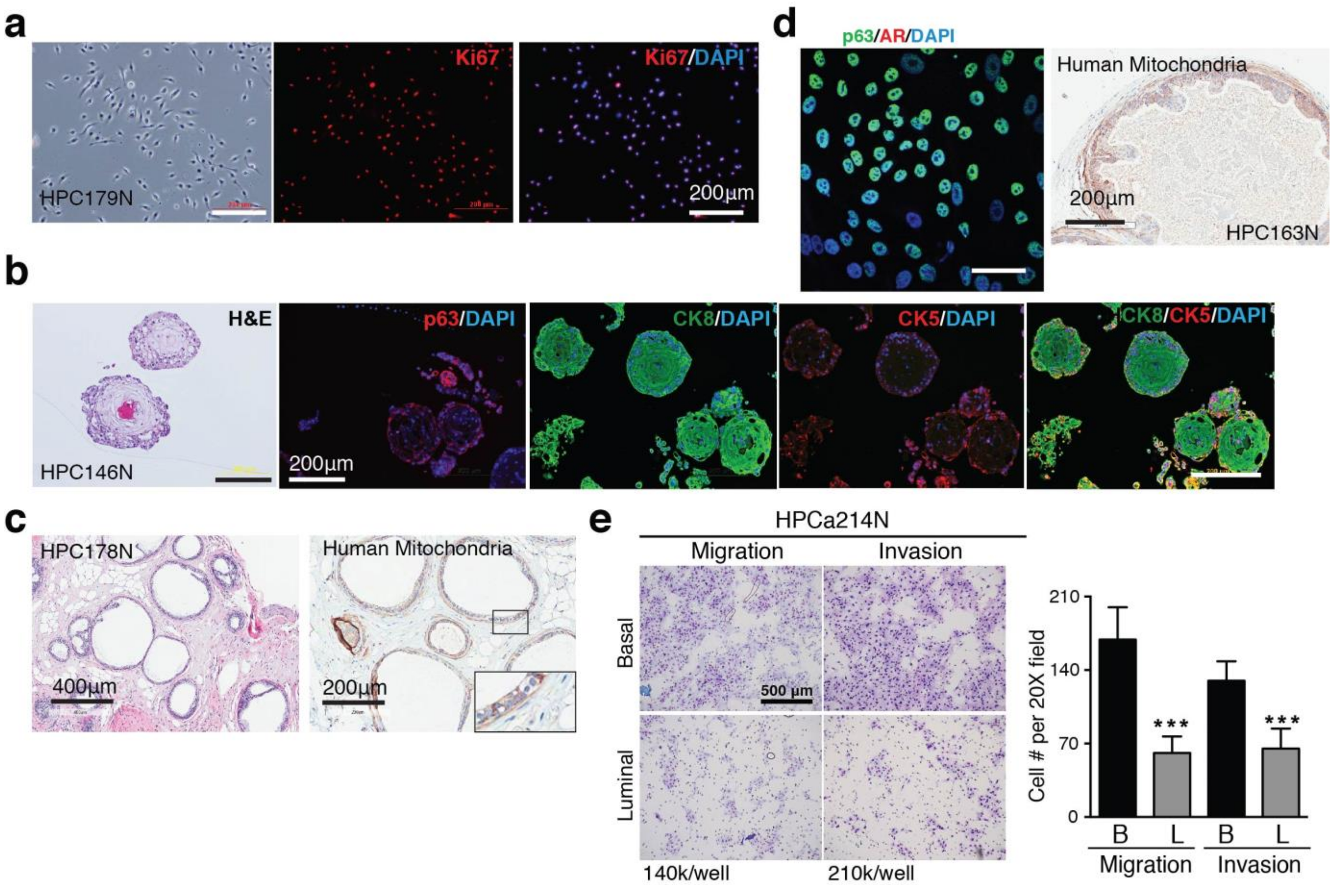


**Supplementary Figure 2.** Human prostatic basal cells exhibit SC properties in vitro and in vivo. **(a)** Ki-67 IF staining in primary HPCa179N basal cell cultures showing the high proliferative fraction. Images were taken from the same field. **(b)** H&E staining and IF analysis of p63, CK5 and CK8 in prostate organoids generated in vitro from primary HPCa146N basal cells. **(c)** IF analysis of p63 and AR identifies the basal cell identity of primary basal cells used for in vivo tissue recombination assay in Fig. 2F (left), and human-specific mitochondrial staining to validate human origin of the regenerated prostatic tissues (right). **(d)** Repeat experiment for Fig. 2F using primary basal cells derived from another benign prostate sample (HPCa178N). Shown are H&E and human-specific mitochondrial staining in prostate tissues regenerated in vivo. **(e)** Boyden chamber migration and invasion assays using primary basal cells freshly purified from another benign prostate tissue sample (HPCa214N). FACS-purified cells at the indicated number were seeded in chambers and the cultures were analyzed 48 h later. Representative low magnification images (left) and quantifications (right) are shown. Data represent the means ± SD from cell number counting of 5~6 random high magnification (20X) images. Results shown (mean ± SD, HPCa214N) were representative data of at least 2~3 repeat experiments

in different patient-derived cell populations. The *P* value was calculated using Student's *t*-test ***P < 0.001. Scale bars, 200μm (a, b, c-right, d); 400μm (c-left), 500μm (e).

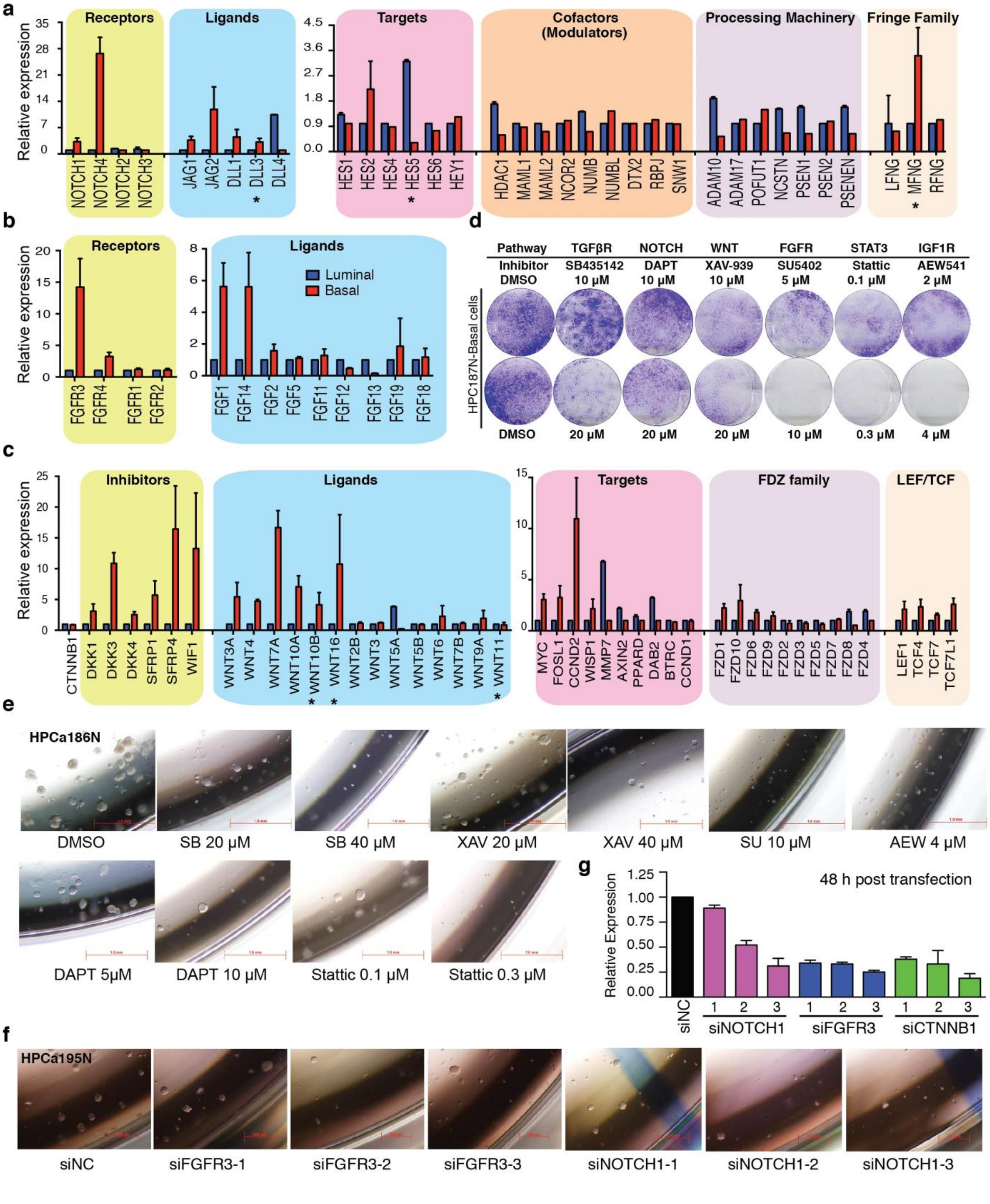

a
Receptors
Ligands
Targets
Cofactors (Modulators)
Processing Machinery
Fringe Family
Relative expression
NOTCH1 NOTCH4 NOTCH2 NOTCH3
JAG1 JAG2 DLL1 DLL3 DLL4
HES1 HES2 HES4 HES5 HES6 HEY1
HDAC1 MAML1 MAML2 NCOR2 NUMB NUMBL DTX2 RBPJ SNW1
ADAM10 ADAM17 POFUT1 NCSTN PSEN1 PSEN2 PSENEN
LFNG MFNG RFNG
b
Receptors
Ligands
Luminal
Basal
FGFR3 FGFR4 FGFR1 FGFR2
FGF1 FGF14 FGF2 FGF5 FGF11 FGF12 FGF13 FGF19 FGF18
d
Pathway TGFβR NOTCH WNT FGFR STAT3 IGF1R
Inhibitor SB435142 DAPT XAV-939 SU5402 Stattic AEW541
DMSO 10 μM 10 μM 10 μM 5 μM 0.1 μM 2 μM
HPC187N-Basal cells
DMSO 20 μM 20 μM 20 μM 10 μM 0.3 μM 4 μM
c
Inhibitors
Ligands
Targets
FDZ family
LEF/TCF
CTNNB1 DKK1 DKK3 DKK4 SFRP1 SFRP4 WIF1
WNT3A WNT4 WNT7A WNT10A WNT10B WNT16 WNT2B WNT3 WNT5A WNT5B WNT6 WNT7B WNT9A WNT11
MYC FOSL1 CCND2 WISP1 MMP7 AXIN2 PPARD DAB2 BTRC CCND1
FZD1 FZD10 FZD6 FZD9 FZD2 FZD3 FZD5 FZD7 FZD8 FZD4
LEF1 TCF4 TCF7 TCF7L1
e
HPCa186N
DMSO
SB 20 μM
SB 40 μM
XAV 20 μM
XAV 40 μM
SU 10 μM
AEW 4 μM
DAPT 5μM
DAPT 10 μM
Stattic 0.1 μM
Stattic 0.3 μM
g
48 h post transfection
Relative Expression
siNC
siNOTCH1
siFGFR3
siCTNNB1
f
HPCa195N
siNC
siFGFR3-1
siFGFR3-2
siFGFR3-3
siNOTCH1-1
siNOTCH1-2
siNOTCH1-3

**Supplementary Figure 3.** Convergent signaling pathways regulate human prostate basal stem/progenitor activity.

**(a-c)** Bar graph presentation of RNA-Seq data showing the expression of signaling components in the Notch (**a**), FGF (**b**), and WNT pathways (**c**) in human prostate basal cells (red bars) and luminal cells (blue bars) relative to each other. Asterisks indicate low gene expression (FPKM<0.32). **(d)** Repeat experiment for Fig. 3b using primary basal cells derived from another benign prostate sample (HPCa187N). Note dose-dependent inhibitory effects of specific signaling pathway inhibitors on colony formation of HPCa187N basal cells. **(e)** Representative images of DMSO vehicle- and inhibitor-treated prostate spheres originated from basal cells. **(f)** Representative images of prostate spheres originated from basal cells infected with control siRNA or siRNAs targeting *FGFR3* and *NOTCH1*. **(g)** qRT-PCR analysis to validate the knockdown efficiency of each siRNA on its target gene.

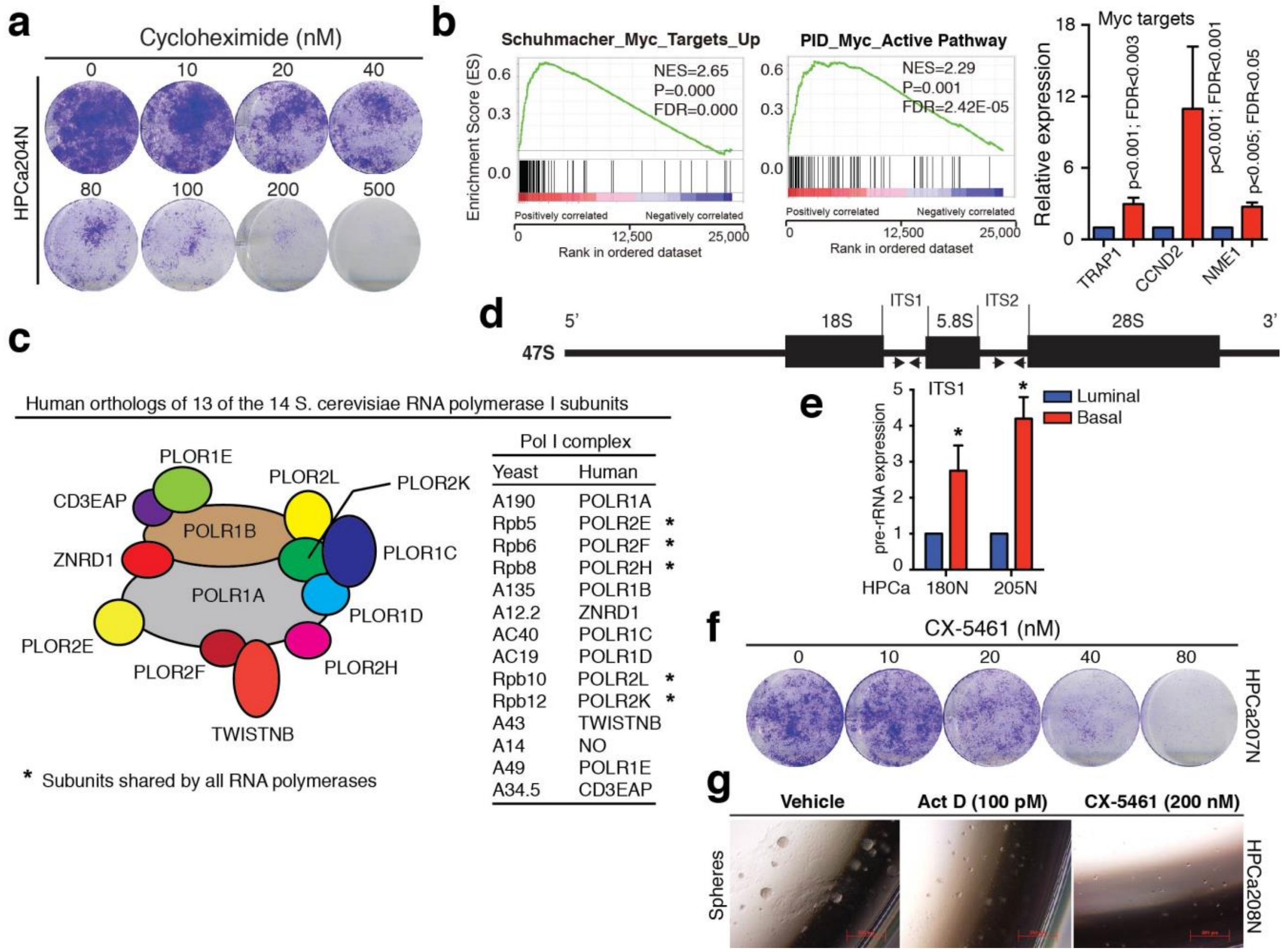


**Supplementary Figure 4.** Enhanced MYC transcriptional program and Pol I-mediated rRNA transcription in basal cells.

**(a)** Protein biosynthesis inhibitor Cycloheximide (CHX) inhibits HPCa204N basal cell proliferation. **(b)** GSEA of two MYC signatures and overexpression of Myc-targets showing an active MYC transcription program in prostatic basal cells. **(c)** Schematic of human RNA polymerase I (Pol I) subunit composition. **(d)** Schematic of the human 47S pre-rRNA structure. After transcription, the large 47S pre-rRNA is converted to the 45S pre-rRNA followed by a serial of processing to finally generate mature 18S, 5.8S and 28S rRNA. qRT-PCR analysis with the primers (black arrows) against the two internally transcribed spacers (ITS1 and ITS2) were used to indirectly measure the rRNA synthesis rate by Pol I. **(e)** qRT-PCR analysis of ITS1 transcripts in primary basal and luminal cells purified from 2 benign samples. The *P* value was calculated using Student's *t*-test *P < 0.05. **(f)** Repeat experiment for Fig.4g in primary basal cells from another benign sample (HPCa207N). **(g)** Inhibitory effects of Actinomycin D (Act. D) and CX-5461 on sphere formation of primary HPCa208N basal cells.

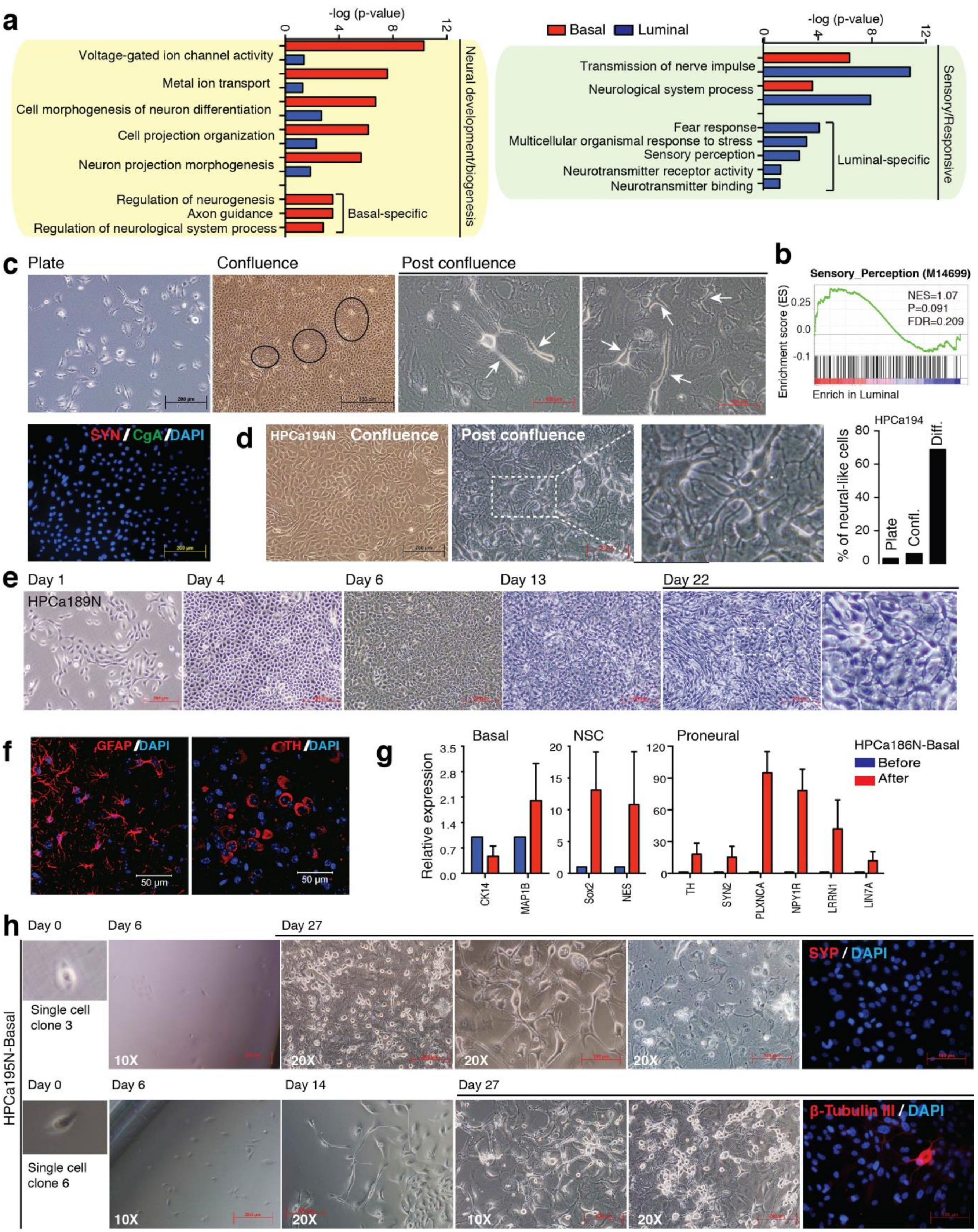

a
-log (p-value)
Basal
Luminal
Voltage-gated ion channel activity
Metal ion transport
Cell morphogenesis of neuron differentiation
Cell projection organization
Neuron projection morphogenesis
Regulation of neurogenesis
Axon guidance
Regulation of neurological system process
Basal-specific
Neural development/biogenesis
Transmission of nerve impulse
Neurological system process
Fear response
Multicellular organismal response to stress
Sensory perception
Neurotransmitter receptor activity
Neurotransmitter binding
Luminal-specific
Sensory/Responsive
b
Sensory_Perception (M14699)
Enrichment score (ES)
NES=1.07
P=0.091
FDR=0.209
Enrich in Luminal
c
Plate
Confluence
Post confluence
SYN / CgA / DAPI
d
HPCa194N
Confluence
Post confluence
HPCa194
% of neural-like cells
Plate
Confl.
Diff.
e
Day 1
Day 4
Day 6
Day 13
Day 22
HPCa189N
f
GFAP / DAPI
TH / DAPI
50 µm
g
Basal
NSC
Proneural
HPCa186N-Basal
Before
After
Relative expression
CK14
MAP1B
Sox2
NES
TH
SYN2
PLXNCA
NPY1R
LRRN1
LIN7A
h
HPCa195N-Basal
Day 0
Day 6
Day 14
Day 27
Single cell clone 3
Single cell clone 6
10X
20X
SYP / DAPI
β-Tubulin III / DAPI

**Supplementary Figure 5.** Prostatic basal cells can spontaneously or be induced to differentiate into neural-like cells.

**(a)** Functional annotation of basal (95 genes) and luminal (71 genes) proneural gene category by DAVID. **(b)** GSEA result for the enrichment of "Sensory perception" gene signature in prostatic luminal cells. **(c)** Morphorlogical changes of in vitro cultured primary basal cells and IF analysis of SYN and CgA (bottom). Arrows indicate cells with typical neural cell morphology. **(d)** Basal cells exhibited typical flat epithelial morphology at confluence, but became neural-like after prolonged culture-induced differentiation. Boxed regions are enlarged. Quantification of morphologically neural-like cells at three different stages of culture was shown in the bar graph (right). **(e)** Repeat experiment for Fig. 5f using primary basal cells from another benign prostate sample (HPCa189N). The default "spontaneous" protocol (see Methods) was utilized. **(f)** IF analysis of GFAP and TH on mouse brain tissues to validate the specificity of the antibodies used. **(g)** Repeat experiment for Fig. 5i using primary basal cells freshly purified from another benign prostate sample (HPCa186N). qRT-PCR analysis of basal cell or NSC markers and a panel of neural/neuronal genes in basal cells before and after proneural differentiation. The relative transcript abundance was normalized to GAPDH levels. **(h)** Single cell clonal analysis showing the proneural differentiation ability of HPCa195N basal cells. Results for two single basal cell generated clones are shown. After prolonged culture of ~ 4 weeks, a single basal cell can generate a heterogeneous clone composed of mixture of cells with typical epithelial and neural-like morphology. Negative staining of SYP indicates that the differentiated neural-like cells are not NE cells. IF analysis indicated that only a few cells could differentiate into β -Tubulin III$^+$ neurons, with the majority of them differentiating into GFAP$^+$ neural cells (see also Fig. 5h).

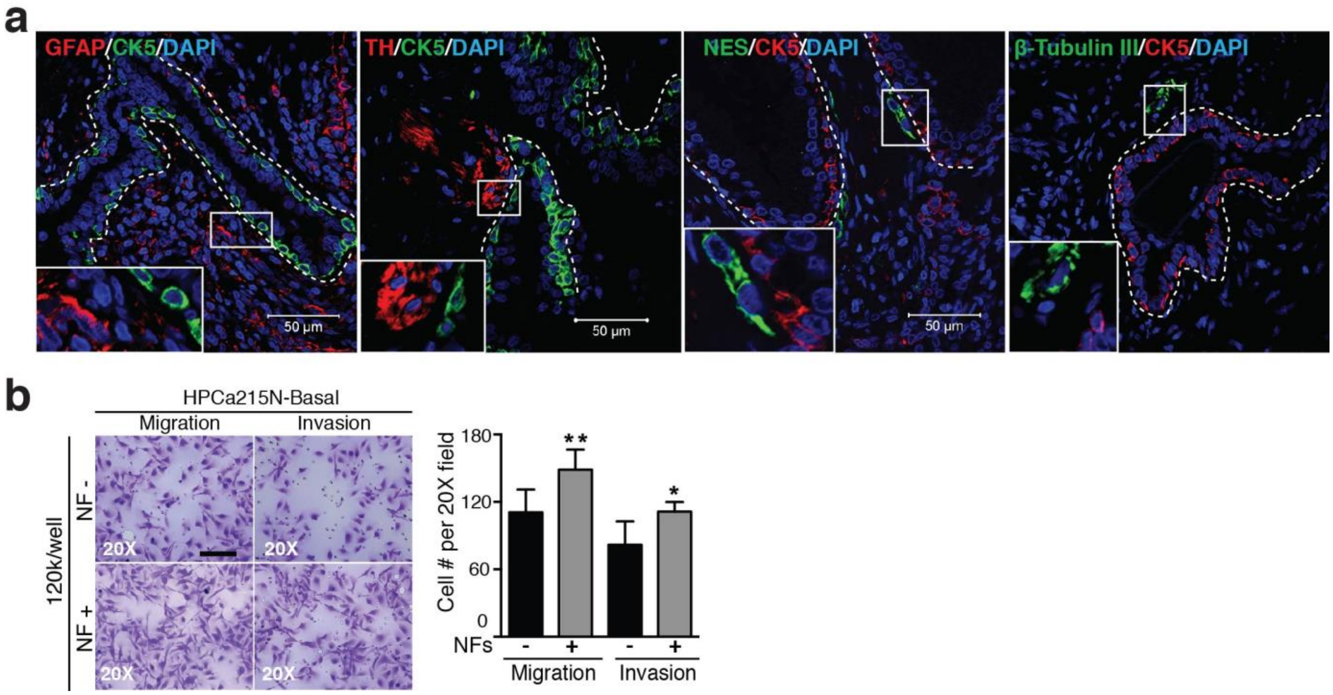


**Figure 6.** Prostate stroma is enriched in neural/neuronal cells and neurotrophic factors regulate migratory/invasive properties of basal cells.

**(a)** Double-immunostaining of CK5 and the indicated neural markers in benign prostate tissues. Scale bars, 50 μm. **(b)** Migration and invasion assays in response to neurotrophic factors (20 ng/ml of BDNF/GDNF/NGF-β, 500 μM GABA, and 0.5 mM db-cAMP). Representative low magnification images (left) and the quantification data (right) are shown. Data represent the mean ± SD from cell number counting of 5~6 random high magnification (20X) images. Results shown (mean ± SD, HPCa215N) were representative data of 2 biological repeat experiments in different patient-derived cell populations. The *P* value was calculated using Student's *t*-test *P < 0.05 and **P < 0.01.

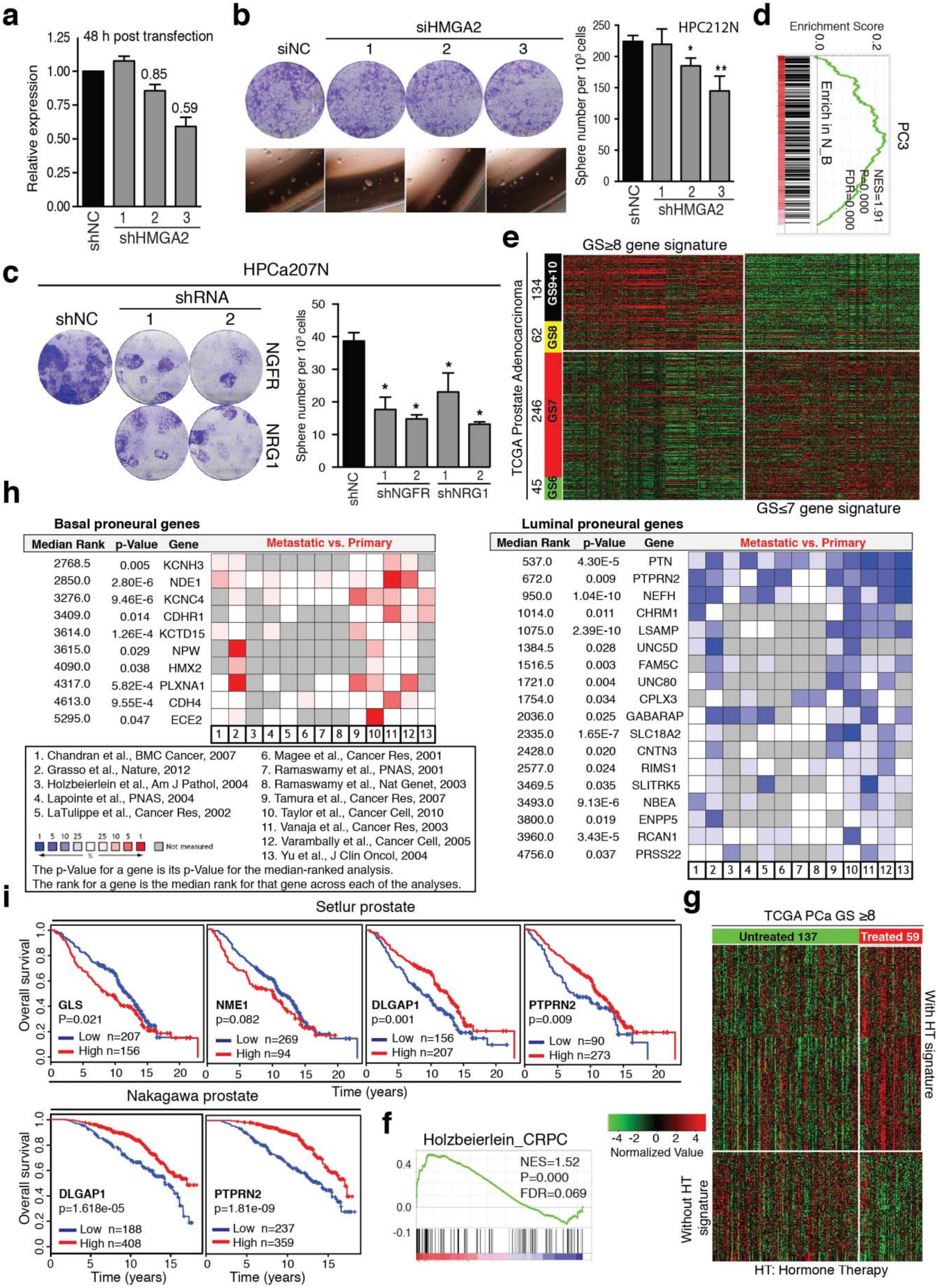
a
48 h post transfection
Relative expression
0.85
0.59
shNC
shHMGA2
b
siNC
siHMGA2
HPC212N
Sphere number per 10^3 cells
shNC
shHMGA2
d
Enrichment Score
Enrich in N_B
PC3
NES=1.91
P=0.000
FDR=0.000
c
HPCa207N
shNC
shRNA
NGFR
NRG1
Sphere number per 10^3 cells
shNC
shNGFR
shNRG1
e
GS≥8 gene signature
TCGA Prostate Adenocarcinoma
134 GS9+10
62 GS8
246 GS7
45 GS6
GS≤7 gene signature
h
Basal proneural genes
Median Rank | p-Value | Gene | Metastatic vs. Primary
2768.5 0.005 KCNH3
2850.0 2.80E-6 NDE1
3276.0 9.46E-6 KCNC4
3409.0 0.014 CDHR1
3614.0 1.26E-4 KCTD15
3615.0 0.029 NPW
4090.0 0.038 HMX2
4317.0 5.82E-4 PLXNA1
4613.0 9.55E-4 CDH4
5295.0 0.047 ECE2
1. Chandran et al., BMC Cancer, 2007
2. Grasso et al., Nature, 2012
3. Holzbeierlein et al., Am J Pathol, 2004
4. Lapointe et al., PNAS, 2004
5. LaTulippe et al., Cancer Res, 2002
6. Magee et al., Cancer Res, 2001
7. Ramaswamy et al., PNAS, 2001
8. Ramaswamy et al., Nat Genet, 2003
9. Tamura et al., Cancer Res, 2007
10. Taylor et al., Cancer Cell, 2010
11. Vanaja et al., Cancer Res, 2003
12. Varambally et al., Cancer Cell, 2005
13. Yu et al., J Clin Oncol, 2004
Not measured
The p-Value for a gene is its p-Value for the median-ranked analysis.
The rank for a gene is the median rank for that gene across each of the analyses.
Luminal proneural genes
Median Rank | p-Value | Gene | Metastatic vs. Primary
537.0 4.30E-5 PTN
672.0 0.009 PTPRN2
950.0 1.04E-10 NEFH
1014.0 0.011 CHRM1
1075.0 2.39E-10 LSAMP
1384.5 0.028 UNC5D
1516.5 0.003 FAM5C
1721.0 0.004 UNC80
1754.0 0.034 CPLX3
2036.0 0.025 GABARAP
2335.0 1.65E-7 SLC18A2
2428.0 0.020 CNTN3
2577.0 0.024 RIMS1
3469.5 0.035 SLITRK5
3493.0 9.13E-6 NBEA
3800.0 0.019 ENPP5
3960.0 3.43E-5 RCAN1
4756.0 0.037 PRSS22
i
Setlur prostate
Overall survival
GLS P=0.021 Low n=207 High n=156
NME1 p=0.082 Low n=269 High n=94
DLGAP1 p=0.001 Low n=156 High n=207
PTPRN2 p=0.009 Low n=90 High n=273
Time (years)
Nakagawa prostate
DLGAP1 p=1.618e-05 Low n=188 High n=408
PTPRN2 p=1.81e-09 Low n=237 High n=359
Time (years)
f
Holzbeierlein_CRPC
NES=1.52
P=0.000
FDR=0.069
Normalized Value
g
TCGA PCa GS ≥8
Untreated 137
Treated 59
With HT signature
Without HT signature
HT: Hormone Therapy

**Supplementary Figure 7** Proneural genes regulate basal stem cell activity and proneural differentiation, and are linked to PCa development.

**(a)** qRT-PCR analysis to validate the knockdown efficiency of each *HMGA2*-siRNA. Primary HPCa195N basal cells were used. **(b)** Repeat experiment for Fig.6a,b using primary basal cells from another benign prostate sample (HPCa212N). Knocking down *HMGA2* by siRNAs in basal cells inhibited colony (left) and sphere (right) formation. The *P* value was calculated using Student's *t*-test $*P < 0.05$ and $**P < 0.01$. **(c)** Repeat experiment for Fig. 5d,e using primary cells from another benign prostate sample (HPCa207N). Knocking down *NGFR* and *NRG1* by shRNA in basal cells inhibited colony (left) and sphere (right) formation. **(d)** GSEA showing the enrichment of PC3 gene signature in human benign prostatic basal cells. **(e)** Clustering analysis of whole-genome transcriptomes of TCGA PCa samples (487 patients) revealing gene signatures corresponding to patients with Gleason Score (GS) under or above 8, respectively. See METHODS for details. **(f)** GSEA showing the enrichment of a CRPC signature in human benign prostatic basal cells. **(g)** Gene clustering analysis of 196 TCGA PCa samples with GS ≥ 8 revealing gene signatures derived from patients with or without hormone therapy (HT). **(h)** Oncomine concept analysis of two proneural gene sets showing that many of the basal and luminal proneural genes are positively and negatively associated with metastatic versus localized primary PCa, respectively. **(i)** Meta-analysis showing higher levels of basal proneural gens (*GLS* and *NME1*) and lower levels of luminal proneural genes (*DLGAP1* and *PTPRN2*) correlating with reduced overall patient survival, respectively. Data were based on the Setlur and Nakagawa studies.

**Supplementary Table 1. Primary benign human prostate tissue samples used in the current study***

| HPC # | Date | Age | GS | Experiments | Related Figures |
|---|---|---|---|---|---|
| HPCa146N | 9/20/12 | 56 | 7 | Sphere formation, Cell culture | Supplementary Fig. 2b; Repeat exp. for Fig. 2e |
| HPCa147N | 9/24/12 | 67 | 7 | Cell culture (Population doubling) | Repeat exp. for Fig. 2e; data not shown |
| HPCa153N | 11/12/12 | 72 | 7 | qRT-PCR on sorted 4 populations, RNA content | Fig. 4b; Supplementary Fig.1b |
| HPCa156N | 12/3/12 | 66 | 7~9 | qRT-PCR on sorted 4 populations | Repeat exp. for Suppl. Fig. 1b; data not shown |
| HPCa163N | 2/18/13 | 62 | 7 | qRT-PCR on sorted 4 populations, Cell culture, TR | Fig. 2f; Repeat exp. for Suppl. Fig. 1b |
| HPCa165N | 2/25/13 | 70 | 7 | qRT-PCR on sorted 4 populations | Repeat exp. for Suppl. Fig. 1b; data not shown |
| HPCa167N | 3/13/13 | 67 | 6 | Colony/sphere formation assay | Fig. 2d; Repeat exp. for Fig. 2c; data not shown |
| HPCa173N | 6/24/13 | 62 | 7 | RNA-Seq, Tissue IF | Fig. 1, 2g, 3b, 4e |
| HPCa175N | 7/15/13 | 67 | 7 | RNA-Seq, Tissue IF, RNA content | Fig. 1, 2g, 3b, 4b, 4e |
| HPCa177N | 7/29/13 | 74 | 6 | RNA-Seq, Tissue IF | Fig. 1, 2g, 3a, 3b, 4e |
| HPCa178N | 8/5/13 | 68 | 9 | Cell culture, TR | Supplementary Fig. 2d |
| HPCa179N | 8/19/13 | 68 | 6 | Cell culture (Population doubling), IF of Ki67, TR | Fig. 2e; Supplementary Fig. 2a; Repeat exp. for Fig. 2f; data not shown |
| HPCa180N | 9/09/13 | 56 | 7 | qRT-PCR of rRNA transcription | Fig. 2c & 4f; Supplementary Fig. 4e |
| HPCa182N | 10/21/13 | 64 | 8 | Cell culture, TR | Repeat exp. for Fig. 2f and Supplementary Fig. 2d; data not shown |
| HPCa186N | 12/2/13 | 65 | 6~7 | Colony/sphere formation (Inhibitors), IF of NSC markers | Fig. 3b, 3c, & 5e; Supplementary Fig. 5g |
| HPCa187N | 12/9/13 | 42 | 6 | Colony/sphere formation (Inhibitors) | Supplementary Fig. 3d |
| HPCa189N | 12/30/13 | 61 | 6~7 | Neural differentiation assay | Supplementary Fig. 5e |
| HPCa190N | 1/6/14 | 64 | 7 | RNA content | Fig. 4b |
| HPCa193N | 1/27/14 | 64 | 6 | RNA content | Fig. 4b |
| HPCa194N | 2/17/14 | 69 | 7~9 | Neural differentiation assay | Fig. 5f, 5g, & 5h; Supplementary Fig. 5d |
| HPCa195N | 3/31/14 | 63 | 6~7 | siRNA exp., Neural differentiation assay (qPCR) | Fig. 3e & 3f; Fig. 4i & 6a-b; Suppl. Fig. 3f & 5h |
| HPCa200N | 6/23/14 | 59 | 6 | Neurosphere assay | Repeat exp. for Fig. 5d; data not shown |
| HPCa202N | 7/7/14 | 61 | 7 | Neurosphere assay | Fig. 5d |
| HPCa204N | 9/8/14 | 68 | 7 | Colony formation (CHX and JQ1 treatment)<br>qRT-PCR of rRNA transcription | Fig. 4f & 4h(left); Supplementary Fig. 4a<br>Repeat exp. for Fig. 3b; data not shown |
| HPCa205N | 9/29/14 | 60 | 6 | Colony formation for pathway inhibitors | Fig. 4f; Supplementary Fig. 4e |

| | | | | | |
|---|---|---|---|---|---|
| | | | | qRT-PCR of rRNA transcription | Repeat exp. for Fig. 3b; data not shown |
| HPCa207N | 10/27/14 | 59 | 7~8 | Colony/sphere assay, Luminal differentiation, JQ1 treatment | Fig. 3g, 6c & 4h(right); Supplementary Fig. 4f & 7c |
| HPCa208N | 11/3/14 | 72 | 7~8 | siRNA and shRNA, Luminal differentiation | Fig. 3e-g, 4g, 4i, 6a-b & 6g; Supplementary Fig. 4g. |
| HPCa212N | 1/26/15 | 70 | 7 | Boyden chamber assay, siRNA exp. | Fig. 2h; Supplementary Fig. 7b |
| HPCa214N | 3/2/15 | 59 | 6~7 | Boyden chamber assay, Cell culture, TR | Supplementary Fig. 2e; Repeat exp. for Suppl. Fig. 6b; data not shown |
| HPCa215N | 3/9/15 | 68 | 6~7 | Boyden chamber assay, Cell culture | Supplementary Fig. 6b |

*A total of 30 patient derived benign prostate tissues were used in this study. These benign tissues were based on negative biopsies (see Supplementary Fig. 1a for examples). The exact utilization of each sample in different figures is also labeled in the panels or indicated in the figure legends. Normally, prostatic epithelial cells were freshly FACS-purified from dissociated tissues according to the expression of CD49f and Trop2, and were then used in various functional studies including proliferation, clonal, sphere, differentiation, knock-down and tissue recombination assays. GS, gleason score.

**Supplementary Table 2. List of 99 genes associated with neural/neuronal function**

| Gene name | Gene symbol | Note |
|---|---|---|
| **Neural and Neuronal Channel/Transporter** | | |
| Potassium channel, subfamily K, member 1 | KCNK1 | |
| Potassium voltage gated channel, subfamily G, member 1 | KCNG1 | |
| Solute carrier family 22, member 17 | SLC22A17 | |
| Solute carrier family 7, member 8 (LAT2) | SLC7A8 | |
| Potassium inwardly-rectifying channel, subf.J, member 8 | KCNJ8 | |
| Solute carrier family 19 member 4 (MCT4) | SLC16A4 | |
| Solute carrier family 16, member 6 (MCT6) | SLC16A6 | |
| Chloride Channel 3 | CLCN3 | |
| Potassium inwardly-rectifying channel, subf. J, member 2 | KCNJ2 | |
| Potassium inwardly-rectifying channel, subf. J, member 2 | KCNJ5 | |
| Potassium inwardly-rectifying channem, subf. J, member 15 | KCNJ15 | |
| **Synaptic Differentiation/Transmission** | | |
| Cortactin | CTTN | |
| Pro-melanin-concentrating hormone | PMCH | |
| Phosphodiesterase E4 | PDE4B | |
| Proprotein convertase subtilisin/kexin type 1 | PCSK1 | |
| Calcium/calmodulin-dependent serine protein kinase | CASK | |
| Glutamate receptor, ionotrophic, AMPA3 | GRIA3 | |
| Synaptotagmin binding, cytoplasmic RNA interacting protein | SYNCRIP | |
| Golgi transport 1 homolog B | GOLT1B | |
| sSnaptotagmin II | SYT2 | |
| Synaptotagmin VIII | SYT8 | |
| Synaptotagmin X | SYT10 | |
| Synaptotagmin-like 4 | SYTL4 | |
| Fibroblast growth factor 7 | FGF7 | |
| Rho GTPase activating protein 6 | ARHGAP6 | |
| Glial fibrillary acidic protein | GFAP | Astrocyte marker |
| Microtubule-associated protein 2 | MAP2 | Neuronal differentiation marker |

| | | |
|---|---|---|
| Tubulin, beta 3 class III | TUBB3 | Nueron marker |
| Tubulin, beta 3 class V | TUBB6 | |
| **Neural and Neuronal Development** | | |
| Nestin | NES | Neural stem cell marker |
| Paired box 6 | PAK6 | Neural stem cell marker |
| SRY (sex determining region Y)-box 2 | SOX2 | Neural stem cell marker |
| Nerve growth factor | NGF | Neuron growth factor |
| Brain derived neurotrophic factor | BDNF | Neuron growth factor |
| Glial cell derived neurotrophic factor | GDNF | Neuron growth factor |
| Neurotrophin 3 | NTF3 | Neuron growth factor |
| Neurotrophin 4 | NTF4 | Neuron growth factor |
| Nerve growth factor receptor | NGFR | Neuron growth receptor |
| Neurotrophic tyrosine kinase, receptor, type 1 | NTRK1 | Neuron growth receptor |
| Neurotrophic tyrosine kinase, receptor, type 3 | NTRK3 | Neuron growth receptor |
| Neurotrophic tyrosine kinase, receptor, type 2 | NTRK2 | Neuron growth receptor |
| Sonic hedgehog | SHH | Neural tube patterning |
| Neuropilin (BRP) and tolloid (TLL)-like 2 | NETO2 | |
| Neuronal growth regulator 1 | NEGR1 | |
| Neuregulin 1 | NRG1 | |
| Netrin G1 | NTNG1 | |
| Noggin | NOG | |
| cadherin 2, type 1, N-cadherin (neuronal) | CDH2 | |
| Platelet-derived growth factor beta polypeptide | PDGFB | |
| Slit homolog 3 (Drosophila) | SLIT3 | |
| Slit homolog 2 (Drosophila) | SLIT2 | |
| Guanine nucleotide binding protein (G protein), alpha activating activity polypeptide, olfactory type | GNAL | |
| Guanine nucleotide binding protein (G protein), alpha inhibiting activity polypeptide 1 | GNAI1 | |
| ADAM metallopeptidase with thrombospondin type 1 motif, 8 | ADAMTS8 | |
| EPH receptor B1 | EPHB1 | |
| GLI family zinc finger 1 | GLI1 | |
| ADAM metallopeptidase domain 8 | ADAM8 | |

| | |
|---|---|
| Secreed frizzled related protein 1 | SFRP1 |
| Acyl-CoA Synthetase long chain family member 3 | ACSL3 |
| Integral membrane protein 2B | ITM2B |
| Low density lipoprotein receptor-related protein 8, apolipoprotein e receptor | LRP8 |
| Neuropilin 2 | NRP2 |
| Sphingomyelin phophodiesterase 1, acid lysosomial (acide sphingomyelinase) | SMPD1 |
| Brain abundant, membrane attached signal protein 1 | BASP1 |
| CDK5 regulatory subunit associated protein 2 | CDK5RAP2 |
| Laminin, alpha 3 | LAMA3 |
| Monoaxygenase, DBH-like 1 | MOXD1 |
| Glutamate-cystein ligase, modifier subunit | GCLM |
| GABA(A) receptor associated protein-like 1 | GABARAPL1 |
| Synuclein, alpha interacting protein (synphilin) | SNCAIP |
| Bone morphogenic protein 6 | BMP6 |
| Syntaxin 1A | STX1 |
| Insuline-like growth factor 1 | IGF-1 |
| Insulin-like growth factor 1 | IGF1R |
| **Other neuronal genes** | |
| FK506 binding protein 7 | FKBP7 |
| EPH receptor A4 | EPHA4 |
| Ly6/neurotoxin1 | LYNX1 |
| Neurofilament 3 (150kDa) | NEF3 |
| Galactosamine:polypeptide N-acetylgalactosaminyltransferase-like 2 | GALNTL2 |
| Glutamine-fructose-6-phosphate transaminase 2 | GFPT2 |
| Ets variant gene 1 | ETV1 |
| Proenkephalin | PENK |
| Fatty acid desaturase | FADS |
| Amphiregulin (schwannoma-derived growth factor) | AREG |
| Phospholipase D 1 | PLD1 |
| Acyl-CoA synthetase long-chain family member 4 | ACSL4 |
| Fatty acid synthase | FASN |
| Insulin like growth factor binding protein 4 | IGFBP4 |
| Suppressor of cytokine signaling 2 | SOCS2 |

| | |
|---|---|
| G-protein coupled receptor 125 | GPR 125 |
| Aldo-keto reductaze family 1, member C1 | AKR1C1 |
| FYN oncogene related to SRC, FGR, YES | FYN |
| Harvey rat sarcoma viral oncogene homolog | HRAS |
| p21 protein (Cdc42/Rac)-activated kinase 7 | PAK7 |
| Phospholipase C, beta 2 | PLCB2 |
| Notch 1 | NOTCH1 |
| Notch 4 | NOTCH4 |
| Fibroblast growth factor receptor 3 | FGFR3 |

Note: The list is modified from Tondreau et al., *BMC Genomics* **9**, 166 (2008).

**Supplementary Table 3. Generation of signatures and datasets from published literature**

| Literature | Figures | Description |
|---|---|---|
| Liu et al., *Cancer Res*. **66**, 4011-4019 (2006) | Fig. 6j | Reported a signature over-represented in PCa samples compared to benign prostate tissues |
| Beltran et al., *Cancer Discov*. **1**, 487-495 (2011) | Fig. 6i,l | Profiled 7 NEPC and 30 PCa, and defined signatures specific for each type |
| Tzelepi et al., *Clin Cancer Res*. **18**, 666-677 (2012) | Fig. 6m | Compared the expression profiles of SCPC and LCNEC xenografts to those of typical prostate adenocarcinoma xenografts, and identified a SCPC/LCNEC signature |
| Irshad et al., *Sci Transl Med*. **5**, 202ra122 (2013) | Fig. 6n | Used a bioinformatics approach to establish a 19-gene signature predictive of indolent PCa with beneficial prognosis |
| Rajan et al., *Eur Urol*. **66**, 32-39 (2014) | Fig. 6p,q | Profiled 7 patients with locally advanced or metastatic PCa, and identified two gene signatures corresponding to before (Fig. 6p) and after (Fig. 6q) ADT treatment |
| Varambally et al., *Cancer Cell* **8**, 393-406 (2005) | Fig. 6r | Used integrative genomic and proteomic analysis to identify a signature of metastatic progression |
| Holzbeierlein et al., *Am J Pathol*. **164**, 217-227 (2004) | Suppl. Fig. 7f | Used microarray-based genome-wide analysis to identify gene expression changes that occur during androgen ablation therapy (goserelin and flutamide) and in CRPC samples |

PCa, prostate cancer; NEPC, neuroendocrine prostate cancer; SCPC, small cell prostate carcinoma; LCNEC, large-cell neuroendocrine carcinoma; ADT, androgen deprivation therapy; CRPC, castration-resistant prostate cancer.

**Supplementary Table 4. Antibodies and primers used in this study**

| Antibody | Supplier | Catalog. No | Species | Dilution |
|---|---|---|---|---|
| DLL4 | Biolegend | 346502 | Mouse | 1:500 |
| CK8 | Developmental Studies Hybridoma Bank | TROMA-1 | Rat | 1:50 |
| COL17A1 | Thermo Fisher Scientific | PA5-26108 | Rabbit | 1:200 |
| HMGA2 | abcam | ab52039 | Rabbit | 1:200 |
| CK5 | Covance | PRB-1609 | Rabbit | 1:500 |
| CK5 | Leica Biosystems | CK5-L-CE-H | Mouse | 1:500 |
| p63 | Cell Signaling | 4892S | Rabbit | 1:500 |
| Ki67 | Leica Biosystems | NCL-Ki67p | Rabbit | 1:500 |
| GFAP | GeneTex | GTX100850 | Rabbit | 1:200 |
| TH | Cell Signaling | 2792S | Rabbit | 1:200 |
| SOX2 | Chemicon | AB5603 | Rabbit | 1:200 |
| NES | Chemicon | MAB5326 | Mouse | 1:200 |
| PAX6 | eBioscience | 14-9914-80 | Mouse | 1:200 |
| MAP2 | Life Technologies | 13-1500 | Mouse | 1:200 |
| NeuN | Chemicon | MAB377 | Mouse | 1:200 |
| Olig2 | Chemicon (Millipore) | AB9610 | Rabbit | 1:100 |
| β-Tubulin III | Thermo scientific | MA1-19187 | Mouse | 1:100 |
| Synaptophysin | Abcam | ab8049 | Mouse | 1:200 |
| Chromogranin A | NeoMarkers | MS-381-P | Mouse | 1:100 |
| NGFR | LSBio | LS-B3440 | Mouse | 1:500 |
| FGFR3 | abcam | ab53636 | Rabbit | 1:50 |
| CDH13 | antibodies-online | ABIN388205 | Rabbit | 1:200 |
| **Primer Name** | **Sequence (Forward 5'-3')** | **Sequence (Reverse 5'-3')** | | |
| hGAPDH | ACTTTGGTATCGTGGAAGGACT | GCCTTGGCAGCGCCAGTAG | | |
| hB2M | ATGGAGGTTTGAAGATGCC | CTAAGTTGCCAGCCCTCCT | | |
| hβ-Catenin | CCACCCTGGTGCTGACTATC | ATTTACAGGTCAGTATCAAACCAG | | |
| hAR | GAGAAGCCTTAGAATGGGTGG | TGGCTTATGGGATAGGACAAC | | |
| hPSA | GGGAGGGTCTTCCTTTGGCA | ATCTGAGGGTTGTCTGGAGGA | | |
| hCK18 | AACAGCCTGAGGGAGGTGGA | CTGTCCAAGGCATCACCAAG | | |

| | | |
|---|---|---|
| hCK14 | AGGAGATCGCCACCTACCGC | CTGGGCAGCCTCAGTTCTTG |
| hCK5 | CTG GTC CAA CTC CTT CTC CA | GGA GCT CAT GAA CAC CAA GC |
| hCD31 | AGGCTCCCTTGATGGAACTTAG | AGGGCAGGTTCATAAATAAGTG |
| hVIM | GACAGGATGTTGACAATGCGT | GCTCCTGGATTTCCTCTTCG |
| hNES | AGGAGATAGAGAGTCCTGGTC | TCCTACAGCCTCCATTCTTG |
| hSOX2 | ATGTCCCAGCACTACCAGA | CCCTCCCATTTCCCTCGT |
| hPAX6 | GATTTTTGGAAAGCCCAGGTA | AAAAGAGATACGAGGTCATCAG |
| hTH | CAGCCCTACCAAGACCAGAC | CACCTAGCCAATGGCACTCA |
| hSYN2 | GCATAGGTGCCATCTGCAT | AAGGTGCTGCTGGTGGTC |
| hNPY1R | TGTTTCTTCAAAGCAGGTCAA | AAGCAGGAGCGAAAAAGACA |
| hLRRN1 | TGGATTTTGTGAATGGGGTT | GTGGGTCTGGATAGCCTTGA |
| hNRXN1 | CCACTCCTCGTGAAACGAAC | TTCAGGCTTGGGTGACTACC |
| hLIN7A | TGAGGGATTGTAGCTTGTGC | AGCGTCACTTCGGCTCC |
| hMAP1B | GGGTTGATCAGGACCACTGT | TGGGACACAAACCTGATTGA |
| hPLXNC1 | CGAAATATCCAGCCAGTTTTCT | CGTGTTGCAAACTGCAATAAA |
| hGAS7 | GCTGGAATCCCAGGAGAACT | AGAAGAAAGCCAGACGGTCA |
| hFGFR3 | GTGTGCAGGTTCCGATGTTATT | CAGGCAAGCAAGGGACAGC |
| hNOTCH1 | GACCGCAGCCCAGTTCCT | GCGGGCGATCTGGGACTG |
| hHMGA2 | CTTCAGCCCAGGGACAACC | CTCCAGTGGCTTCTGCTTTCT |
| hCD49f | GTGTTGGGAGGGTGGTTCA | CCCCGAATCCCATTGCTTTG |
| hCD3EAP | CCCAGTGCTGGGATTATAGGT | GAGGTCAAGGCAGGTGGATC |
| hMYC | CGGTTTTCGGGGCTTTATCTAAC | AGTTTCGTGGATGCGGCAAG |
| h47S rRNA ITS1 | GACCCCTTGGGGGGATCG | CGCGGACACCACCCCACA |
| h47S rRNA ITS2 | CCCGCCCCGCGGCCCGC | CGACGCGGAAGCTCGGGA |
| hCDH13 | GTAAAAGCGGAAAGTTAGTGC | ACCTTACCCTAGTTCGTGGC |
| hNRG1 | CTACATCTACATCCACCACTGG | ACTTGCACAAGTATCTCGAGG |
| hNGFR | GCCTGAAGTTGGAGTGAGTG | GCCATTTACTACAGTGCTCCTA |
| hp63 | AATCAACTTTGTGGGTGGAGAG | TAGTAGGTCTTCCAGGGTTTCA |

Note: “h” means human.

**Supplementary Table 5. The sequences of siRNA and shRNA used in this study**

| Name | Company | Catalog. No | Sequence siRNA | Species |
|---|---|---|---|---|
| FGFR3 siRNA | OriGene | SR301584 | ACCUCGACUACUACAAGAAGACAAC | Human |
| | | | AGGUUGUUAAUAGUUGGAGGUGATT | |
| | | | GGUUUAUUCCGGAAACUAGUGUACA | |
| HMGA2 siRNA | OriGene | SR305363 | GCAAGACUCAGGAGCUAGCAGCCCG | Human |
| | | | ACAAGAGUCCCUCUAAAGCAGCUCA | |
| | | | CCAAGCCGCUUCCGAAGUGCUCCCG | |
| NOTCH1 siRNA | OriGene | SR303207 | CCUGCAAAGACAUGACCAGUGGCTA | Human |
| | | | AGUGAAAGCAUAUGGGUUAGAUGTT | |
| | | | GGAAGUUGAACGAGCAUAGUCCAAA | |
| CD3EAP siRNA | OriGene | SR307394 | GCAAUACAGCGAAUCAGCGGCUUTC | Human |
| | | | GGCGUUGCAACAAACCAUAUUGGAC | |
| | | | AGCACAGCCCUACUAACUAGUAUTC | |
| CTNNB1 siRNA | OriGene | SR301063 | GGAUCACAAGAUGGAAUUUAUCAAA | Human |
| | | | CGCAUGGAAGAAAUAGUUGAAGGTT | |
| | | | AGAAUUGAGUAAUGGUGUAGAACAC | |
| CDH13 siRNA | OriGene | SR300727 | UGCAUACAAAGAGGUGUACAGGUAC | Human |
| | | | AGUGGAUCAAGAGCCUAAAGGAATT | |
| | | | CCCUGAAUGUUUAAAGAUCAUGACA | |
| NRG1 shRNA | GE Dharmacon | Clone ID: V2LHS_84774 | ATATACTCTCCAGAATCAG | Human |
| | | Clone ID: V3LHS_344002 | TCTTGAACCACTTGAATCT | Human |
| NGFR shRNA | GE Dharmacon | V2LHS_152261 | TTTCCCAGAAGGTCACCTG | Human |
| | | V2LHS_152259 | TATGACACCTGCTGTGGTG | Human |

Note: For siRNA and shRNA experiments, 3 siRNAs and 2 shRNAs targeting the same gene were used, respectively.

## Supplementary Discussion

**Integrated SC, EMT, neurogenic and rRNA biogenesis programs in basal cells confer intrinsic SC properties and developmental plasticity**

Our deep RNA-Seq analysis revealed very distinct transcriptomes of human benign prostatic basal and luminal cells (Fig.1). Although RNA-Seq was done in highly purified cells from 3 pairs of benign samples, related biological studies and functional characterizations were conducted in more than a dozen of samples. Basal cells preferentially express gene signatures enriched in ESCs and mammary SCs. Numerous developmental and SC genes (e.g., SHH, HMGA2, SOX2, MYC, and genes in WNT, NOTCH, and FGF/IGF/TGFβ signaling) are all preferentially expressed in basal cells. Supporting the functional importance of these genes, pharmacological inhibition of the pathways or genetic ablation of representative genes impair the proliferative, clonal, and sphere-forming capabilities of primary basal cells. Many of these genes and signaling pathways also regulate EMT. In fact, basal cells are enriched in EMT gene signatures as well as several core EMT-regulating transcription factors. Strikingly, >11% of all genes expressed in basal cells have neurogenic roles such as promoting neural development, axonal guidance, and neural progenitor functions. Consistent with this gene expression profile, primary basal cells can spontaneously or be induced to undergo 'neural' development in vitro, generating NSC-like cells. In this context, it is interesting to note that when ESCs undergo spontaneous differentiation, their 'default' cell fate is towards the neural lineage[1]. Presumably, preferential expression of EMT molecules and gene signatures, coupled with neurogenic gene expression and NSC-like features, endow basal cells high migratory and invasive capabilities. Basal cells, like ESCs and other SCs[2-4] also show increased total RNA contents, Pol II-mediated transcription, and, in particular, Pol I-associated rRNA biogenenesis. Knocking down representative genes in Pol I complex diminishes both proliferative and clonogenic properties of primary basal cells. Heightened Pol I activity and rRNA synthesis may be associated with preferential expression of MYC and MYC transcriptional program in basal cells, as supported by studies with JQ1. Combined, these transcriptional programs are integrated to endow some basal cells not only intrinsic SC properties but also lineage plasticity. This latter point is also buttressed by genetic studies in the mouse prostate that demonstrate the developmental plasticity of basal cells[5].

**Crosstalk between basal and luminal cells and between epithelial cells and stroma**

Exhaustive annotations of basal and luminal gene expression profiles, coupled with relevant biological assays, suggest that there may exist extensive cross communications between the two epithelial lineages as well as between epithelia and the underlying ECM and stroma (Fig. 7). Analysis of the NOTCH, WNT, FGF, and TGFβ pathways suggests that basal cells engage both autocrine and paracrine signaling whereas luminal cells mainly produce ligands to engage paracrine signaling (Fig. 7a). Simultaneous expression of many signaling receptors and activating ligands implies a cell-autonomous enforcement of stemness in basal cells. In support, inhibition of NOTCH and FGFR not only reduces proliferative and sphere-forming properties but also enhances differentiation of basal cells. In the FGF/FGFR pathway, basal cells specifically express FGFR3/4 and several ligands but luminal cells highly express FGF13 (Fig. 7b), again suggesting that basal cells both self-regulate in an autocrine manner (i.e., self-sustenance) and also receive paracrine signals from luminal cells. On the other hand, luminal cells specifically express DLL4 (Fig. 1i), suggesting that the luminal cells secret ligands to regulate basal cells (Fig. 7a). Intriguingly, basal cells express not only many WNT-activating ligands but also multiple WNT inhibitors (Fig. 2b), raising the possibility that WNT signaling plays a key 'rheostat' role in maintaining the relative quiescence of basal cells (Fig. 2g) whereas keeping them poised for differentiation induced by demand and environmental cues. In support, disrupting this rheostat inhibits proliferative and clonogenic properties and also promotes differentiation.

Gene annotations and biological studies also suggest cross communications between epithelial cells and the ECM and stromal components (Fig. 7b,c). To our surprise, as much as 7.5% of the luminal cell-specific genes can also be classified as proneural genes. Remarkably, however, the luminal proneural genes are mostly involved in neuronal signal reception and processing. Considering that the prostate is a richly innervated organ[6] and the prostate stroma has readily detectable cells that express neural/neuronal markers such as GFAP, TH, NES, and β-tubulin III (Supplementary Fig. 6a), it is tempting to speculate that luminal cells, and perhaps some basal cells, are constantly communicating with the nervous system in the stroma to rapidly respond to microenvironmental changes. In this regard, prostate epithelial cells resemble post-synaptic target cells that receive and interpret the neural signals (e.g., neuropeptides, neurotransmitters, and neurotrophic factors; Fig. 7b) from neural/neuronal cells in the stroma. In addition,

the immediate juxtaposition of basal cells to the stroma, which is enriched in TGF-β[7], and preferential expression of TGFBR2 in basal cells (Fig. 2b) may help regulate their relative dormancy. Moreover, basal cells may produce their own ECM components that function together with the stromal ECM and stromal cells to establish a SC niche for the basal SCs (Fig. 7c). A prototypical example could be COL17A1, which is among the most highly enriched genes in basal cells (Fig. 1i) and has been shown to play a crucial role in maintaining the niche of bulge keratinocyte stem cells[8].

**Transcriptional profiles of epithelial cell types linked to PCa clinical features: relevance to understanding PCa cell-of-origin and developing novel PCa therapies**

One of the most significant findings here that has clear-cut clinical relevance is that the basal gene expression profile can be linked to aggressive PCa subtypes and adverse patient outcomes. Specifically, basal cell gene expression profile resembles those in high-grade primary tumors and anaplastic PCa (including a spectrum of undifferentiated variants)[9,10]. Anaplastic PCa normally occurs in 1–2% of men with PCa; however up to 10–20% of cases are found on repeat biopsies during CRPC progression. Importantly, the basal cell gene expression profile is also enriched in CRPC and associated with metastasis and poor patient overall survival. Strikingly, previous studies have shown that the collective invasion in breast cancer requires a conserved basal epithelial program[11]. In support, a recent study indicated that human metastatic breast cancer cells possess a basal/stem-like transcriptional program[12]. More relevantly, many SC-related pathways (e.g., Wnt) are upregulated in advanced PCa after ADT[13]. Moreover, our findings are consistent with the observations in colorectal cancer where gene expression profiles of tumors have been shown to resemble those of the normal epithelial lineages and the signatures of normal epithelial subsets are of predictive value of disease progression[14]. Our results also suggest that the basal cell gene expression profile or signatures may be developed into a “biomarker” for aggressive PCa with poor prognosis.

Of clinical importance, the molecular resemblance of basal cells to anaplastic PCa and CRPC provides a common molecular understanding of these diverse and poorly characterized aggressive PCa subtypes. Understanding the distinct gene expression profiles in basal versus luminal cells also sheds fresh lights on the etiology of both adenocarcinomas and variant PCa (Fig. 7d). As the luminal layer is self-sustained by proliferating progenitors[15,16] and luminal cells seem to be overall more proliferative[5] (Fig. 2g), luminal cells

could, in theory, be more susceptible to tumorigenic transformation[17]. On the other hand, SCs in the basal layer possess long-term proliferative potential (Fig. 2e) and, likely, longer lifespan, and hence could function as the cells-of-origin for adenocarcinomas upon oncogenic insult-triggered differentiation[15] as well as for the small subset of $AR^{-/lo}$ anaplastic PCa variants (Fig. 7d). CRPC, on the other hand, may represent a mixture of expanded basal-like undifferentiated tumor cells and de-differentiated luminal cells caused by persistent castration[18-20].

In principle, genes critically important for maintaining the basal cell stemness may also operate in anaplastic and castration-resistant PCa cells and thus constitute therapeutic targets. These may include both well-established genes such as HMGA2, MYC, and SOX2 as well as novel molecules such as COL17A1 and CDH13. Increased transcription of rRNA genes by Pol I is a common feature of human cancer. Our data also reveal an important role of active rRNA transcription, mediated via a MYC transcriptional program, in maintaining the SC properties of basal cells. MYC is a key regulator of rRNA transcription and a MYC network accounts for similarities between ESCs and cancer cell transcription programs[21]. This connection may suggest a rationale for treating anaplastic PCa and CRPC with Pol I inhibition[22,23]. Along this line, we have obtained preliminary evidence that CX-5461 demonstrates significant therapeutic efficacy against experimental CRPC (Zhang et al., unpublished data). It was previously thought that *MYC* amplification and/or MYC overexpression is a late event only in advanced PCa. Recent studies, however, indicate that *MYC* amplification also occurs in a subset of primary localized PCa[24] and MYC protein is overexpressed in the majority of precursor lesions and PCa[25]. Together, our results reinforce the concept that MYC and the MYC-mediated transcriptional program represent critical therapeutic targets in PCa.

## Supplementary References


1. Kamiya, D. *et al.* Intrinsic transition of embryonic stem-cell differentiation into neural progenitors. *Nature* **470**, 503-509 (2011).
2. Efroni, S. *et al.* Global transcription in pluripotent embryonic stem cells. *Cell Stem Cell* **2**, 437-447 (2008).
3. Watanabe-Susaki, K. *et al.* Biosynthesis of ribosomal RNA in nucleoli regulates pluripotency and differentiation ability of pluripotent stem cells. *Stem Cells* **32**, 3099-3111 (2014).
4. Zhang, Q., Shalaby, N.A. & Buszczak, M. Changes in rRNA transcription influence proliferation and cell fate within a stem cell lineage. *Science* **343**, 298-301 (2014).
5. Wang, Z.A. *et al.* Lineage analysis of basal epithelial cells reveals their unexpected plasticity and supports a cell-of-origin model for prostate cancer heterogeneity. *Nat. Cell. Biol.* **15**, 274-283 (2013).
6. Magnon, C. *et al.* Autonomic nerve development contributes to prostate cancer progression. *Science* **341**, 1236361 (2013).
7. Salm, S.N. *et al.* TGF-β maintains dormancy of prostatic stem cells in the proximal region of ducts. *J. Cell. Biol.* **170**, 81-90 (2005).
8. Tanimura, S. *et al.* Hair follicle stem cells provide a functional niche for melanocyte stem cells. *Cell Stem Cell* **8**, 177-187 (2011).
9. Beltran, H. *et al.* Aggressive variants of castration-resistant prostate cancer. *Clin. Cancer. Res.* **20**, 2846-2850 (2014).
10. Nadal, R., Schweizer, M., Kryvenko, O.N., Epstein, J.I. & Eisenberger, M.A. Small cell carcinoma of the prostate. *Nat. Rev. Urol.* **11**, 213-219 (2014).
11. Cheung, K.J., Gabrielson, E., Werb, Z. & Ewald, A.J. Collective invasion in breast cancer requires a conserved basal epithelial program. *Cell* **155**, 1639-1651 (2013).
12. Lawson, D.A. *et al.* Single-cell analysis reveals a stem-cell program in human metastatic breast cancer cells. *Nature* **526**, 131-135 (2015).
13. Rajan, P. *et al.* Next-generation sequencing of advanced prostate cancer treated with androgen-deprivation therapy. *Eur. Urol.* **66**, 32-39 (2014).
14. Merlos-Suarez, A. *et al.* The intestinal stem cell signature identifies colorectal cancer stem cells and predicts disease relapse. *Cell Stem Cell* **8**, 511-524 (2011).
15. Choi, N., Zhang, B., Zhang, L., Ittmann, M. & Xin, L. Adult murine prostate basal and luminal cells are self-sustained lineages that can both serve as targets for prostate cancer initiation. *Cancer Cell* **21**, 253-265 (2012).
16. Karthaus, W.R. *et al.* Identification of multipotent luminal progenitor cells in human prostate organoid cultures. *Cell* **159**, 163-175 (2014).
17. Wang, Z.A., Toivanen, R., Bergren, S.K., Chambon, P. & Shen, M.M. Luminal cells are favored as the cell of origin for prostate cancer. *Cell Rep.* **8**, 1339-1346 (2014).
18. Liu, X., Chen, X., Rycaj, K., Chao, H.P., Deng, Q., Jeter, Collene., Liu, C., Honorio, S., Li, H., Davis, T. Systematic dissection of phenotypic, functional, and tumorigenic heterogeneity of human prostate cancer cells. *Oncotarget* **6**, 23959-23986 (2015).
19. Qin, J. *et al.* The PSA(-/lo) prostate cancer cell population harbors self-renewing long-term tumor-propagating cells that resist castration. *Cell Stem Cell* **10**, 556-569 (2012).

20. Schroeder, A. *et al.* Loss of androgen receptor expression promotes a stem-like cell phenotype in prostate cancer through STAT3 signaling. *Cancer Res.* **74**, 1227-1237 (2014).
21. Kim, J. *et al.* A Myc network accounts for similarities between embryonic stem and cancer cell transcription programs. *Cell* **143**, 313-324 (2010).
22. Bywater, M.J. *et al.* Inhibition of RNA polymerase I as a therapeutic strategy to promote cancer-specific activation of p53. *Cancer Cell* **22**, 51-65 (2012).
23. Drygin, D. *et al.* Targeting RNA polymerase I with an oral small molecule CX-5461 inhibits ribosomal RNA synthesis and solid tumor growth. *Cancer Res.* **71**, 1418-1430 (2011).
24. Boutros, P.C. *et al.* Spatial genomic heterogeneity within localized, multifocal prostate cancer. *Nat. Genet.* **47**, 736-745 (2015).
25. Gurel, B. *et al.* Nuclear MYC protein overexpression is an early alteration in human prostate carcinogenesis. *Mod. Pathol.* **21**, 1156-1167 (2008).
